# sDR5-Fc Administered Post-Exposure Alleviates Radiation-Induced Enteric Injury by Inhibiting ROS and NLRP3/Caspase-1/GSDMD-Dependent Pyroptosis and Inflammatory Response

**DOI:** 10.3390/ph19101647

**Published:** 2026-10-19

**Authors:** Xuan Qiao, Shuang Guo, Zhuang Tian, Lin-Tao Li, Yu-Han Yang, Zhi-Yun Meng, Hui Gan, Shu-Chen Liu, Gui-Fang Dou, Ruo-Lan Gu

**Affiliations:** 1 Graduate Collaborative Training Base of Academy of Military Medical Sciences, Hengyang Medical School, University of South China, Hengyang 421001, China; 2 Beijing Institute of Radiation Medicine, Beijing 100850, China

**Keywords:** decoy receptors, sDR5-Fc, pyroptosis, inflammasomes, gasdermin D, oxidative stress, radiation-induced intestinal injury, intestinal mucosal damage

## Abstract

**Background:** The risk of ionizing radiation exposure, including accidents and medical complications, has increased with the rapid development and broad application of nuclear technology. The intestine is highly susceptible to radiation, with radiation-induced intestinal injury characterized by a complex cascade of cellular and molecular reactions. This cascade results in the death of intestinal epithelial cells, which compromises the integrity of the intestinal mucosal barrier and disrupts immune regulation. At present, there are several limitations in the research on the therapy of radiation-induced intestinal injury, including uncertain mechanisms and an absence of optimal pharmaceuticals. This study aims to explore the post-exposure treatment effects of sDR5-Fc (a DR5 antagonist) on radiation-induced intestinal pyroptosis damage. **Methods:** In this study, C57BL/6 mice, human HIEC-6, and rat IEC-6 intestinal epithelial cells were exposed to a single 15 Gy high-dose γ-irradiation to establish an acute radiation-induced intestinal injury model. All sDR5-Fc treatments were administered after radiation exposure to simulate clinical post-exposure intervention. In vivo histological staining was performed for quantitative evaluation of intestinal damage. In vitro cell viability and LDH release assays were used to evaluate intestinal epithelial injury. Multiple detection methods, including scanning electron microscopy (SEM), flow cytometry, peripheral blood routine analysis, organ index detection, immunohistochemistry, qRT-PCR, and Western blotting, were applied to assess radiation-induced alterations in oxidative stress (ROS, MDA) and pyroptosis-associated inflammasome indicators (NLRP3, caspase-1, GSDMD-N, IL-1β, IL-18). **Results:** It was observed that treatment with soluble DR5-Fc fusion protein (sDR5-Fc), a DR5 antagonist, markedly reduced these radiation-induced effects. Mice subjected to 15 Gy total abdominal γ-irradiation exhibited similar outcomes after sDR5-Fc administration, including inhibited pyroptosis in intestinal and colonic tissues, enhanced recovery of peripheral blood cells (leukocytes, erythrocytes, and platelets), improved organ indices of the spleen and thymus, and an increased 30-day survival rate. Histological examination and pathological scoring confirmed significant structural damage in the irradiated group, and these pathological alterations were partially reversed following sDR5-Fc administration. Molecular docking and BLI assays suggest a potential in vitro interaction between DR5 and NLRP3, which can be interfered with by sDR5-Fc. Moreover, qRT-PCR and Western blotting assays indicated that sDR5-Fc attenuated pyroptosis by reducing the NLRP3/caspase-1/GSDMD signaling pathway. **Conclusions:** These findings suggest that sDR5-Fc, as a DR5 antagonist, is a promising therapeutic candidate for mitigating high-dose radiation-induced intestinal injury; however, further research is warranted.

## 1. Introduction

Ionizing radiation is the energy released by atoms in the form of particles (e.g., α-particles, β-particles, electrons, protons, neutrons, etc.) or as electromagnetic waves (e.g., X-rays or γ-rays), sufficient to ionize atoms or molecules [1]. With the rapid advancement of nuclear technology, nuclear energy has been widely utilized in various fields of human society, such as the military, energy production, clinical medicine, industry, and agriculture. Ionization radiation provides significant advantages to human society and economic progress, but it concurrently increases the risk of radiation toxicity for individuals. Exposure to a significant dose of ionizing radiation over a brief duration, whether systemically or locally, can induce acute radiation syndrome (ARS), leading to considerable radiological damage, particularly to the hematological system, intestines, brain, and other organs [2,3,4]. The United States Food and Drug Administration (FDA) has approved drugs including amifostine (WR-2721), fexofenadine, polyethylene glycol fexofenadine, and sargramostim for radiation protection. However, the current clinical efficacy and use of these pharmaceuticals remain limited, possibly attributable to their high cost, narrow therapeutic indices, brief half-lives, limited effectiveness, or substantial adverse effects, etc. [5,6,7].

Biological damage induced by radiation is very complex. Research indicates that ionizing radiation-induced cell death may involve various pathways, including apoptosis [8,9], pyroptosis [10,11], necroptosis [12,13], ferroptosis [14,15], and autophagy [16,17], etc. However, multiple molecular targets and inter-regulatory systems that govern radiation-induced cell death remain inadequately recognized. The digestive system is one of the most susceptible tissues to radiation. Radiation enteritis is a prevalent type of injury resulting from high-dose radiation, typically severe and challenging to treat [18,19,20]. Research has demonstrated that exposure to radiation levels between 6 and 15 Gy significantly damages intestinal tissues, primarily evidenced by the fast degradation of the intestinal crypts and mucosal structure. These alterations may result in mucosal inflammation, bacterial infiltration, and sepsis, hence elevating the infection risk [21]. Radiation-induced mucosal barrier breakdown forms a deleterious positive feedback loop with mucosal immune hyperactivation and endotoxemia: disruption of epithelial integrity allows luminal antigens and bacterial endotoxins to translocate into the lamina propria and systemic circulation, which in turn amplifies local and systemic inflammatory responses and further aggravates barrier injury. Seminal work by Wang et al. identified microvascular endothelial barrier protection as a critical intervention node for breaking this vicious cycle in radiation-induced intestinal injury [22]. From a macro-pathological perspective, Huang et al. established a multi-target framework for intestinal mucosal repair, which provides a theoretical paradigm for synergistically restoring barrier integrity and normalizing immune homeostasis in radiation enteropathy [23]. Currently, there are only a few treatment methods for radiation-induced intestinal injury, mainly comprising symptomatic supportive care, which yields suboptimal outcomes.

Chen et al. indicated that the majority of mice who underwent full-body irradiation (15 Gy) only survived for 4 days post-exposure and had intestinal cell death due to apoptosis, autophagy, and necroptosis [8]. Cao et al. discovered that ionizing radiation induces pyroptosis in both tumor cells and normal intestinal cells [11,24]. Hu et al. recently demonstrated that over 50% of AIM2 and CASP1/11 mutant mice survived for 30 days following abdominal irradiation at 14.2 Gy [25]. These findings indicate that the inflammatory vesicle route, which includes Caspase-1/11, is crucial in modulating intestinal radiosensitivity. Furthermore, Shao Feng’s team confirmed that activated Caspase-1 and caspase-4/5/11 share GSDMD and transfer its N-terminal domain to the cell membrane, followed by cell expansion and cell death [26], suggesting the significance of pyroptosis in radiation-induced cell death.

The TRAIL-DR5 signaling pathway is an essential apoptosis-inducing mechanism within the tumor necrosis factor (TNF) superfamily. Membrane-bound TRAIL binds to cell-surface DR5 to trigger DISC assembly and apoptotic signaling at the plasma membrane; the TRAIL-DR5 complex is subsequently internalized as a regulatory event. In contrast, soluble sDR5-Fc acts as a decoy receptor by sequestering autocrine/paracrine membrane-bound TRAIL, thereby preventing TRAIL from engaging cell-surface DR5 and blocking upstream DISC formation [27]. It functions as a crucial mediator of extrinsic apoptosis and is essential for organismal development, homeostasis, immunological control, and disease progression. DR5 agonists have been comprehensively studied for their therapeutic efficacy in cancer [28,29,30,31]; however, recent findings indicated their upregulation in target organs of other pathologies, including cardiovascular ischemia–reperfusion injury [32,33,34], autoimmune disorders [35,36,37], neurodegenerative diseases [38,39,40], and severe viral infections [41,42]. Moreover, DR5 antagonism can mitigate tissue damage caused by excessive apoptosis. Our previous study revealed that after a single 6 Gy γ-ray irradiation, DR5 expression was upregulated in hematopoietic and immune tissues (e.g., thymus) of mice, causing functional impairment. In addition, the administration of the soluble DR5-Fc fusion protein (sDR5-Fc), a DR5 antagonist, significantly facilitated tissue healing and improved the 30-day survival rate of irradiated mice [27], highlighting DR5 as a potential therapeutic target for radiation-induced injury. This study further validated the efficiency of sDR5-Fc and investigated the mechanisms by which it mitigates oxidative stress and pyroptosis induced by high-dose (15 Gy) single abdominal γ-ray irradiation, via in vivo and in vitro radiation enteritis models.

Based on our previous work demonstrating the radioprotective activity of sDR5-Fc in hematopoietic injury models, the present study aimed to explore the therapeutic potential and underlying mechanism of sDR5-Fc against high-dose γ-ray-induced intestinal injury. Beyond simply characterizing the protective phenotype of sDR5-Fc, the key scientific novelty of this work lies in establishing a previously underappreciated functional link between DR5 and NLRP3/Caspase-1/GSDMD-mediated pyroptosis in radiation-damaged intestinal epithelium. Using in vitro intestinal epithelial cell models and a mouse total-abdominal irradiation model, we demonstrated that post-irradiation administration of sDR5-Fc suppresses oxidative stress, excessive inflammation, and pyroptosis, improves tissue repair, and prolongs animal survival. Distinct from amifostine, which requires prophylactic injection before irradiation, sDR5-Fc exerts therapeutic effects when delivered after radiation exposure, conferring practical advantages for emergency countermeasures against accidental nuclear exposure. Collectively, our findings identify sDR5-Fc as a promising therapeutic candidate for mitigating radiation-induced intestinal injury and provide new mechanistic insights connecting DR5 signaling to inflammasome-driven pyroptosis.

## 2. Results

### 2.1. In Vitro Studies

#### 2.1.1. sDR5-Fc Recovered the Membrane Integrity of Enterocytes After 15 Gy γ-Irradiation

**Figure 1:**
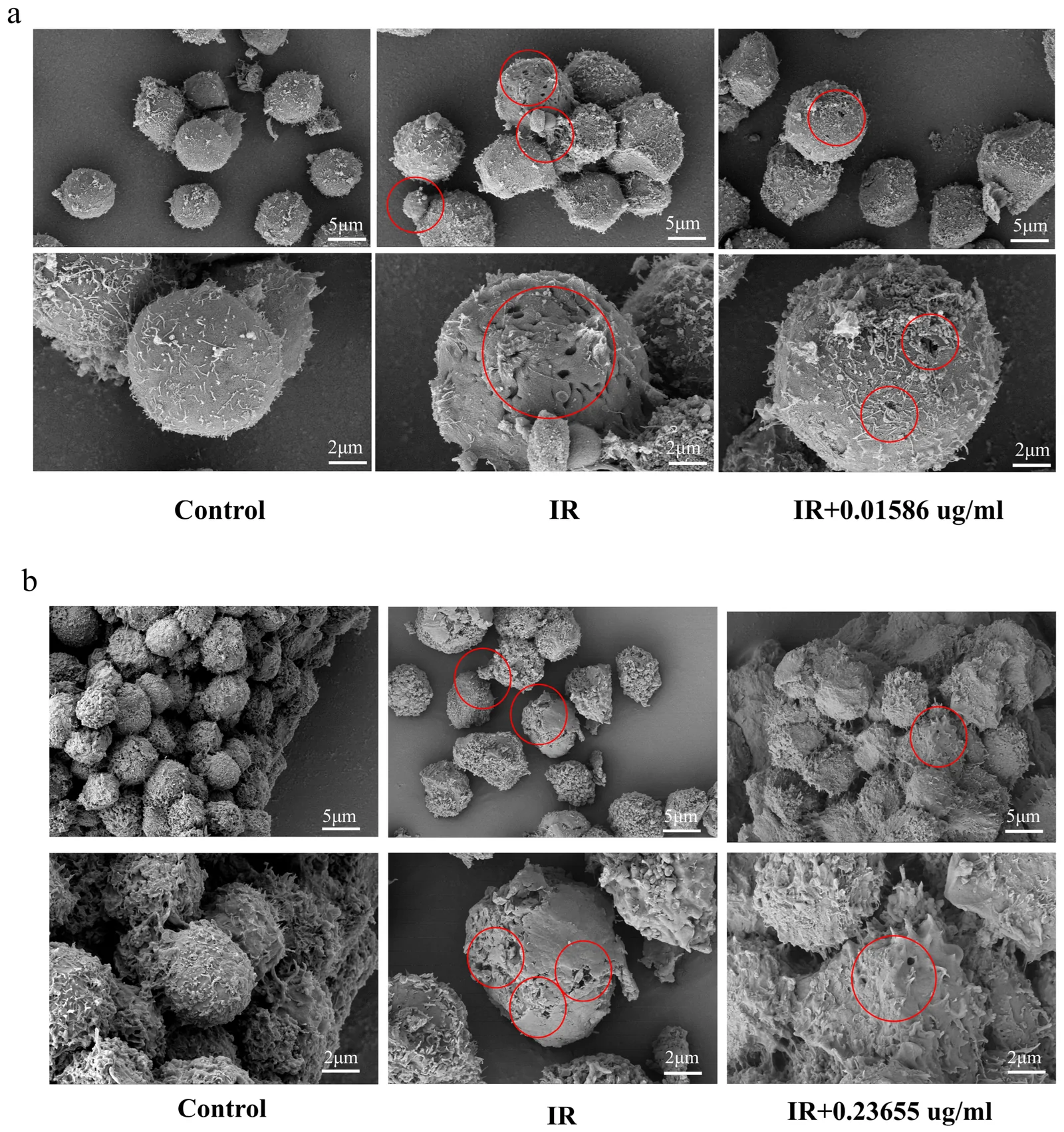
Effect of sDR5-Fc on HIEC-6/IEC-6 cell membranes after irradiation. (**a**) HIEC-6, (**b**) IEC-6. The content highlighted by the red circle in the figure demonstrates the characteristics of pyroptosis.Pyroptotic cells exhibit cell swelling, plasma membrane pore formation and rupture, while the nucleus remains largely intact.

After 24 h of irradiation, compared with the normal group, the model group cells exhibited significant features of pyroptosis, including irregular morphology, bulging cell membranes, and a cell surface characterized by numerous vesicle-like protuberances (pyroclastic vesicles) as well as small holes (Figure 1). Furthermore, relative to the model group, the sDR5-Fc intervention can significantly reduce cell pyroptosis, characterized by fewer and smaller window pores, as well as fewer vesicular protrusions and small pores. This indicates that sDR5-Fc can reduce the formation of pyknotic vesicles and attenuate the IR-induced cell death.

#### 2.1.2. sDR5-Fc Inhibited the Pyroptosis of Enterocytes After 15 Gy γ-Irradiation

**Figure 2:**
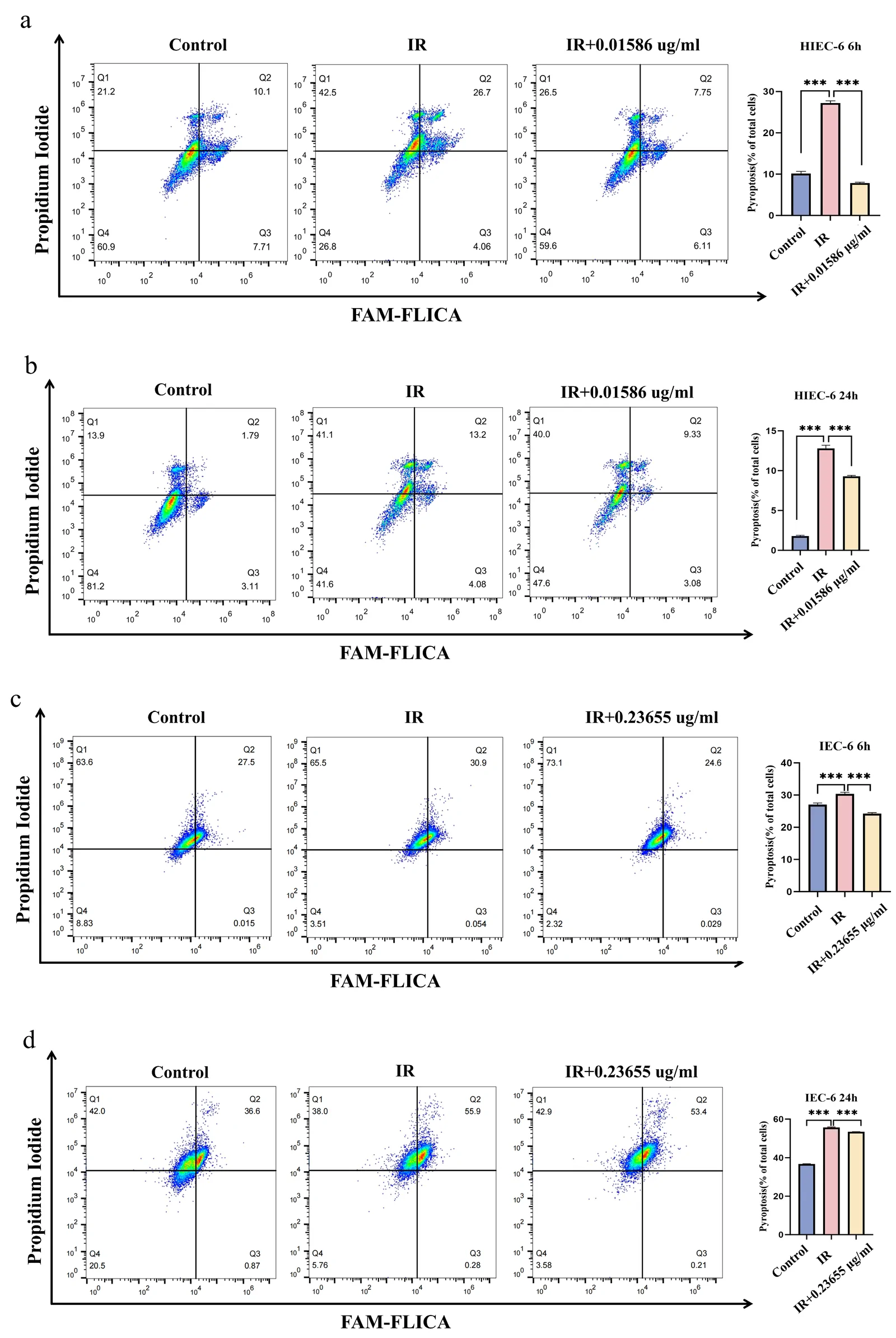
Effect of sDR5-Fc on the pyroptosis of irradiated HIEC-6/IEC-6 cells. (**a**) HIEC-6 at 6 h. (**b**) HIEC-6 at 24 h. (**c**) IEC-6 at 6 h. (**d**) IEC-6 at 24 h. *** *p* < 0.001 vs. IR group.

Flow Cytometry analysis was carried out to assess pyroptosis after irradiation and the protective effect of sDR5-Fc. The protective effect of sDR5-Fc was indicated when pyroptosis occurred after the proportion of FAM-YVAD-FMK and PI double-positive cells (Q2) increased [43]. Relative to the control group, the proportion of double-positive HIEC-6 and IEC-6 cells was significantly increased (*** *p* < 0.001 vs. IR group) at 6 and 24 h after irradiation, respectively, whereas this proportion was reduced after sDR5-Fc administration (*** *p* < 0.001 vs. IR group) (Figure 2).

**Figure 3:**
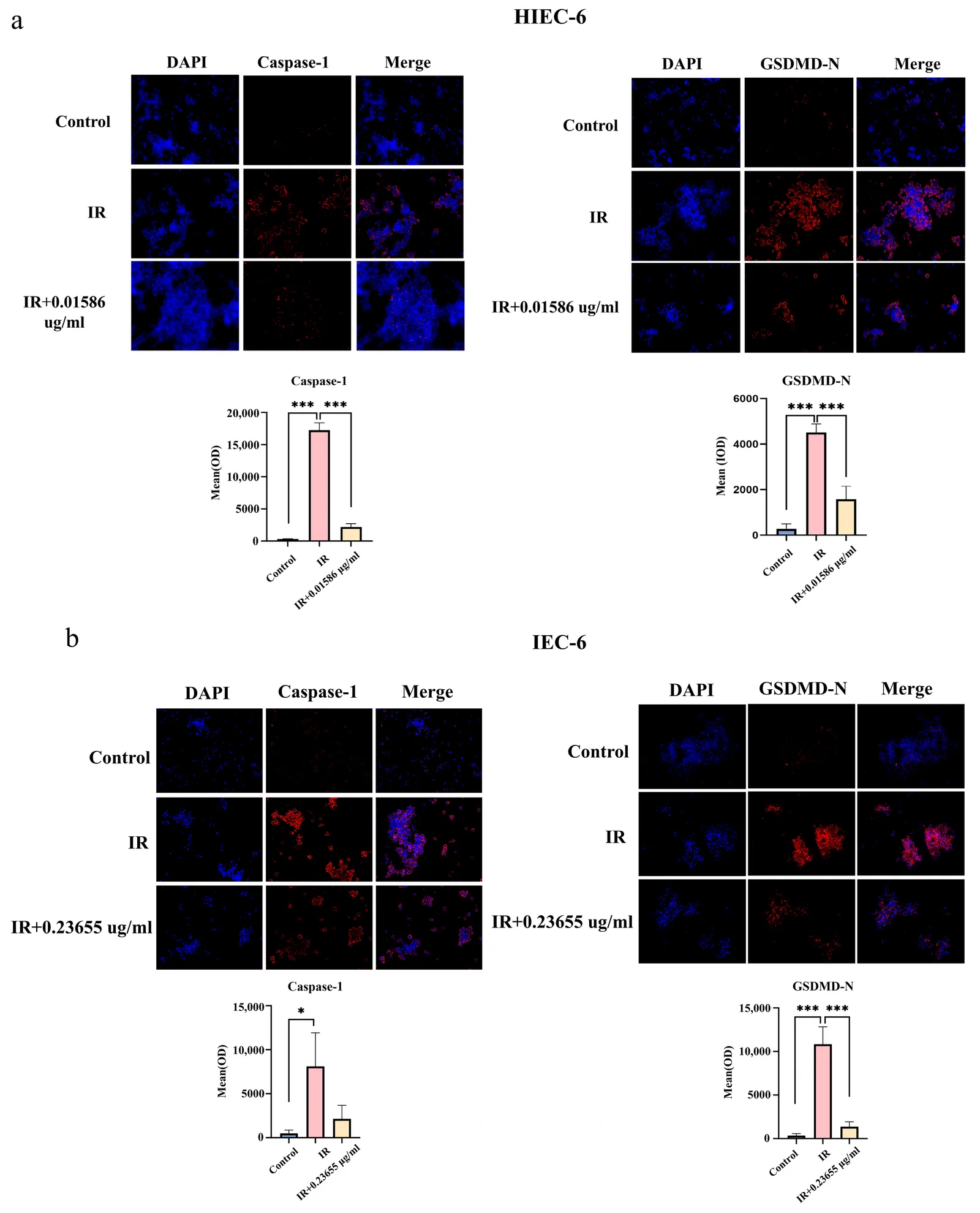
Effect of sDR5-Fc on Caspase-1 and GSDMD-N in irradiated (**a**) HIEC-6 cells and (**b**) IEC-6 cells. * *p* < 0.05, *** *p* < 0.001 vs. IR group.

Immunofluorescence staining revealed (Figure 3) that 15 Gy γ-irradiation significantly increased the fluorescence intensity of Caspase-1 and GSDMD-N in HIEC-6/IEC-6 cells (* *p* < 0.05, *** *p* < 0.001 vs. control group), whereas sDR5-Fc treatment significantly decreased their expression (*** *p* < 0.001 vs. IR group).

#### 2.1.3. sDR5-Fc Inhibited LDH Release from Enterocytes After 15 Gy γ-Irradiation

**Figure 4:**
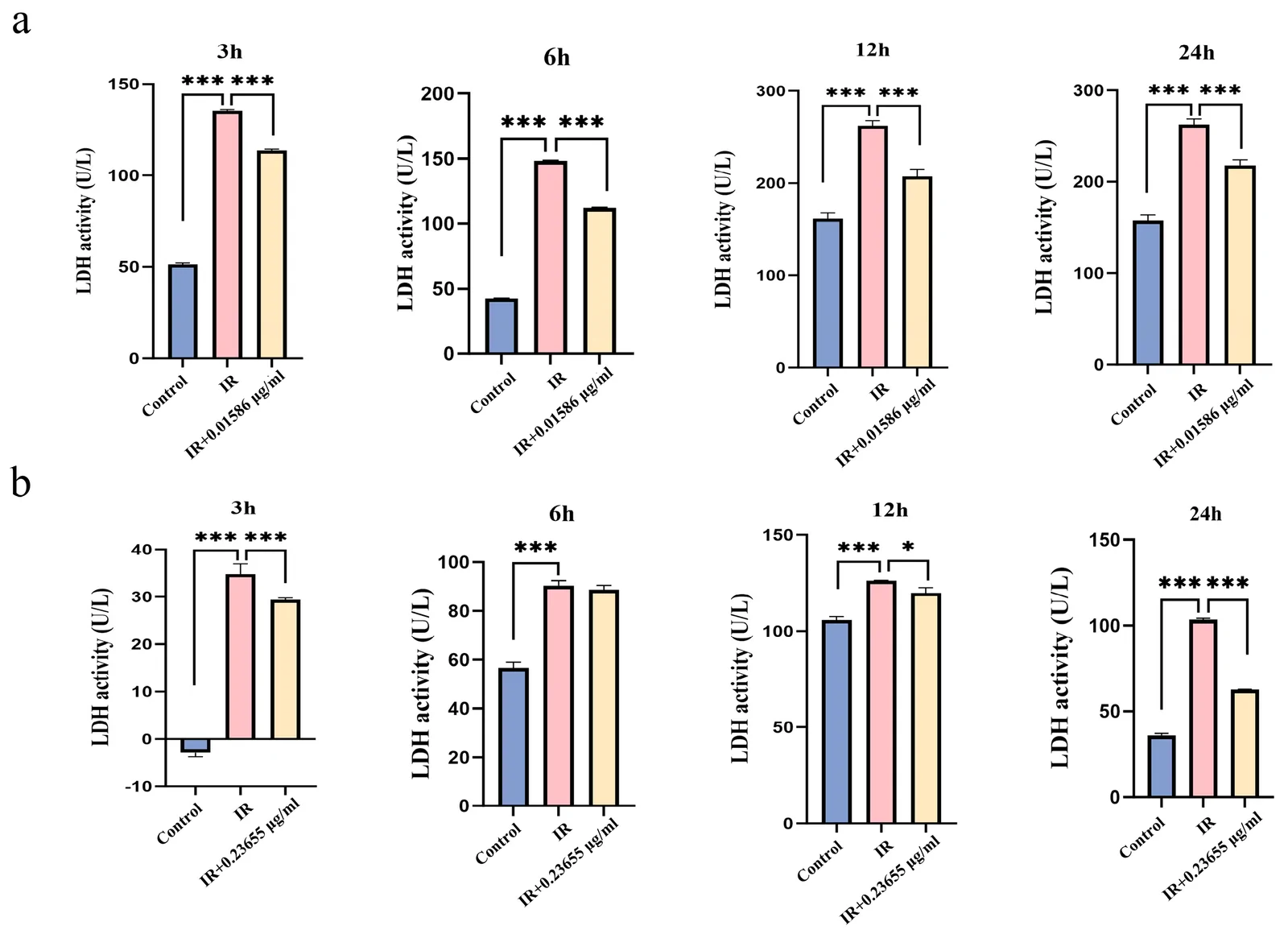
Effect of sDR5-Fc on LDH release in HIEC-6/IEC-6 cellular pyroptosis model. (**a**) HIEC-6; (**b**) IEC-6. * *p* < 0.05, *** *p* < 0.001 vs. IR group.

LDH is a remarkably stable enzyme found in the cytoplasm. Upon cellular injury, intracellular LDH is released into the culture medium. Therefore, cell pyroptosis results in the release of various soluble proteins, including LDH, due to membrane rupture; thus, pyroptosis can be assessed by measuring LDH release [44]. Here, it was observed that in 15 Gy γ-irradiation-exposed HIEC-6 (Figure 4a) and IEC-6 (Figure 4b) cells, the LDH levels were significantly increased in the cell supernatant compared with the normal control cells, at 3, 6, 12, and 24 h, respectively (*** *p* < 0.001 vs. control group) (Figure 4). However, sDR5-Fc intervention significantly reduced these levels (* *p* < 0.05, *** *p* < 0.001 vs. the IR group), indicating that sDR5-Fc administration after irradiation can effectively reduce enterocyte cell damage.

#### 2.1.4. sDR5-Fc Inhibited K^+^ Efflux from Irradiated Enterocytes

**Figure 5:**
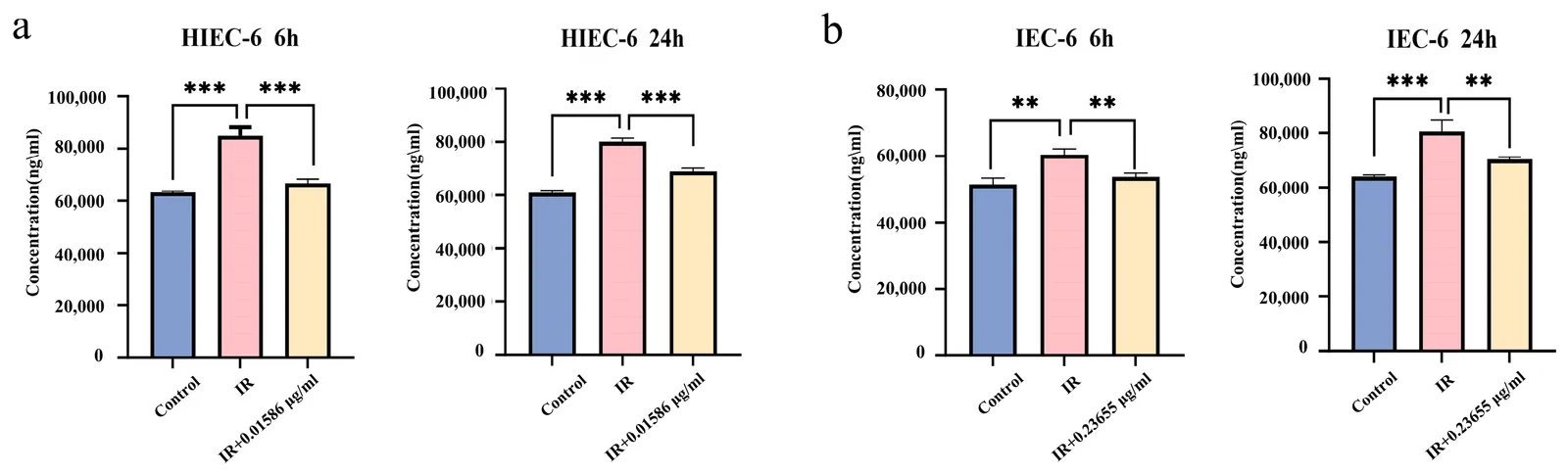
Effect of sDR5-Fc on K^+^ efflux in post-irradiation (**a**) HIEC-6 cells and (**b**) IEC-6 cells. ** *p* < 0.01, *** *p* < 0.001 vs. IR group.

The literature has demonstrated that reduced cytoplasmic K^+^ concentration induces the assembly of NLRP3 inflammatory vesicles. Furthermore, K^+^ efflux is crucial for certain particles to disrupt lysosomal activity and stimulate NLRP3 inflammatory vesicles [45,46,47]. Here, K^+^ efflux was assayed in HIEC-6 cells and IEC-6 cells at 6 h and 24 h, respectively, after 15 Gy γ-irradiation. The results revealed that the extracellular K^+^ concentration was significantly higher in both the irradiated HIEC-6 and IEC-6 cells compared to the normal control (** *p* < 0.01 or *** *p* < 0.001 vs. control group) (Figure 5). However, sDR5-Fc administration reduced extracellular K^+^ concentration in both cell types at 6 and 24 h (** *p* < 0.01 or *** *p* < 0.001 vs. the IR group), suggesting that irradiation caused K^+^ efflux from enterocytes, while sDR5-Fc significantly inhibited K^+^ efflux caused by irradiation.

#### 2.1.5. sDR5-Fc Significantly Reduced the Oxidative Level of IR-Induced Enterocytes

Several studies have shown that ionizing radiation can indirectly harm DNA molecules through direct interaction or by generating reactive oxygen species (ROS), which induce oxidative stress [48,49,50,51]. Excessive ROS leads to oxidative damage. Malondialdehyde is a byproduct of lipid peroxidation [52,53,54]. Oxidative denaturation and elevated ROS in tissues and cells increase MDA levels, which serve as a significant marker for assessing oxidative damage [55,56]. Superoxide dismutase primarily converts superoxide anion radicals into hydrogen peroxide, and radiation can diminish the activity or concentration of SOD in cells or tissues [57,58,59]. To investigate the potential function of sDR5-Fc in radiation-induced oxidative damage, the levels of ROS, MDA, and SOD in irradiated enterocytes treated with sDR5-Fc were assessed.

**Figure 6:**
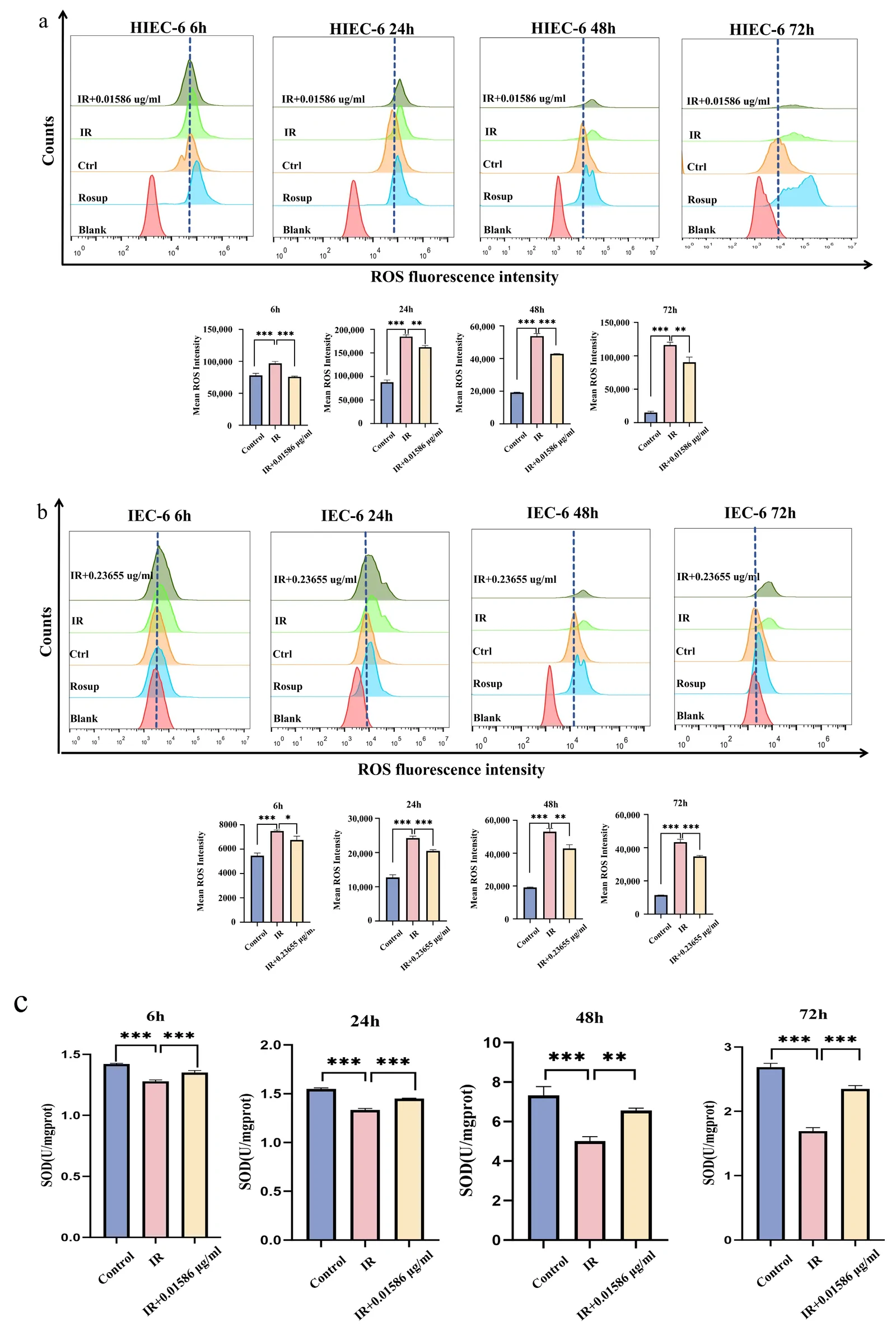

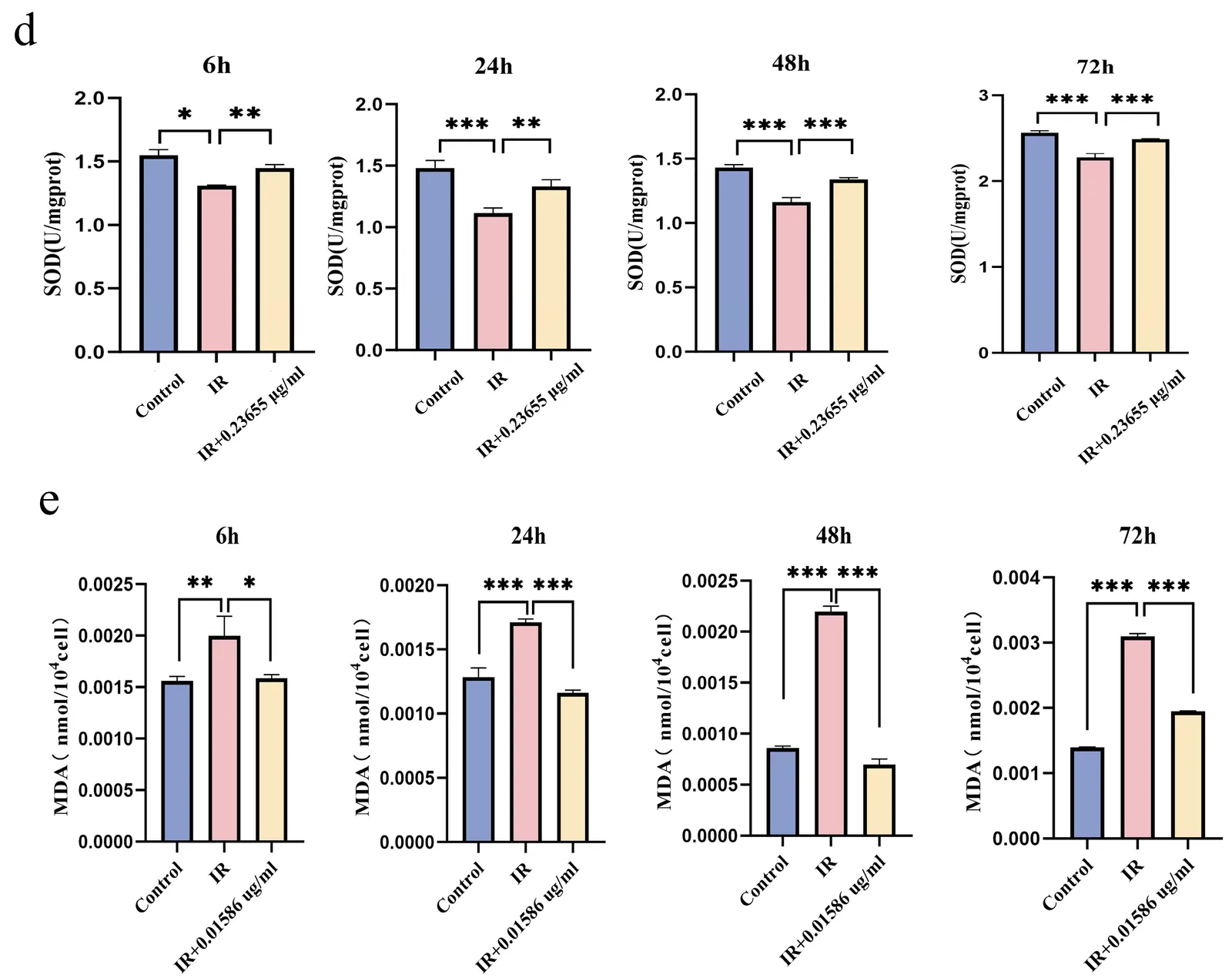
Effect of sDR5-Fc on oxidation levels in HIEC-6/IEC-6 cells after radiation exposure. (**a**) ROS levels in HIEC-6 and (**b**) IEC-6 cells. (**c**) SOD levels in HIEC-6 and (**d**) IEC-6 cells. (**e**) MDA levels in HIEC-6 cells. * *p* < 0.05, ** *p* < 0.01, *** *p* < 0.001 vs. IR group.

The results (Figure 6) showed that after 6, 24, 48, and 72 h of irradiation, both the ROS and MDA levels in enterocytes were significantly increased, whereas sDR5-Fc treatment significantly reduced these levels. Furthermore, SOD levels in enterocytes at 6, 24, 48, and 72 h after irradiation were significantly lower than those of the normal control, whereas sDR5-Fc therapy reversed these changes. These results suggest that 15 Gy γ-irradiation induced oxidative damage in enterocytes, but sDR5-Fc alleviated this radiation-induced oxidative damage.

### 2.2. In Vitro Mechanism Study

#### 2.2.1. DR5 Affinity with NLRP3

**Figure 7:**
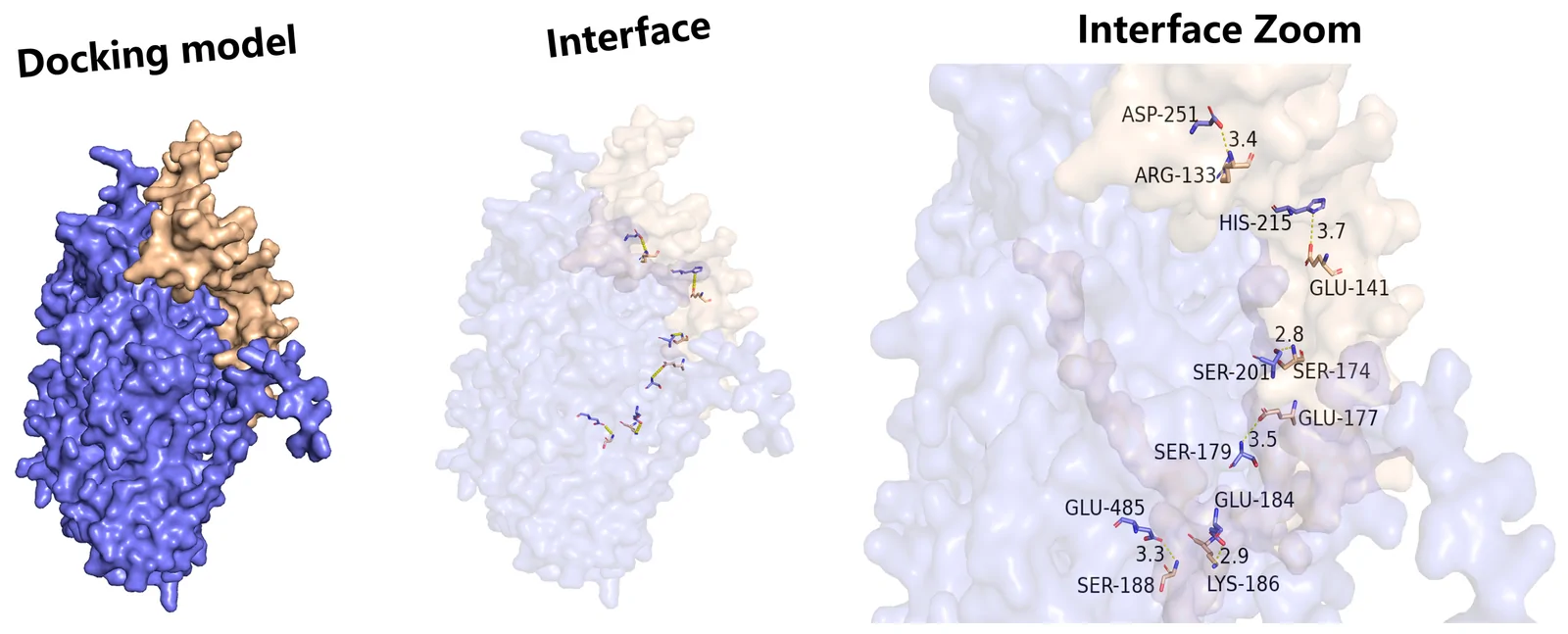
Molecular docking model. Molecular docking of NLRP3 with DR5 (NLRP3: blue; DR5: yellow).

Blind docking analysis was performed between human DR5 and NLRP3 using the molecular docking software GRAMM-X v1.2.0. The data revealed that the free energy of the DR5–NLRP3 interaction was −8.5 kcal/mol (Figure 7). Furthermore, the GLU141 of DR5 formed a hydrogen bond with the HIS215 of NLRP3 (bond length = 3.7 Å), GLU177 of DR5 hydrogen bonded with SER179 of NLRP3 (bond length = 3.5 Å), ARG133 of DR5 hydrogen bonded with ASP251 of NLRP3 (bond length = 3.4 Å), SER174 of DR5 hydrogen bonded with SER201 of NLRP3 (bond length = 2.8 Å), LYS186 of DR5 hydrogen bonded with GLU184 of SER201 (bond length = 2.9 Å), and SER188 of DR5 hydrogen bonded with SER201 of GLU485 (bond length = 3.3 Å). These docking results suggest a potential physical interaction interface between DR5 and NLRP3 in silico, which may participate in the activation of pyroptosis-related signaling.

#### 2.2.2. sDR5-Fc Inhibited the Binding of DR5 to NLRP3 In Vitro

**Figure 8:**
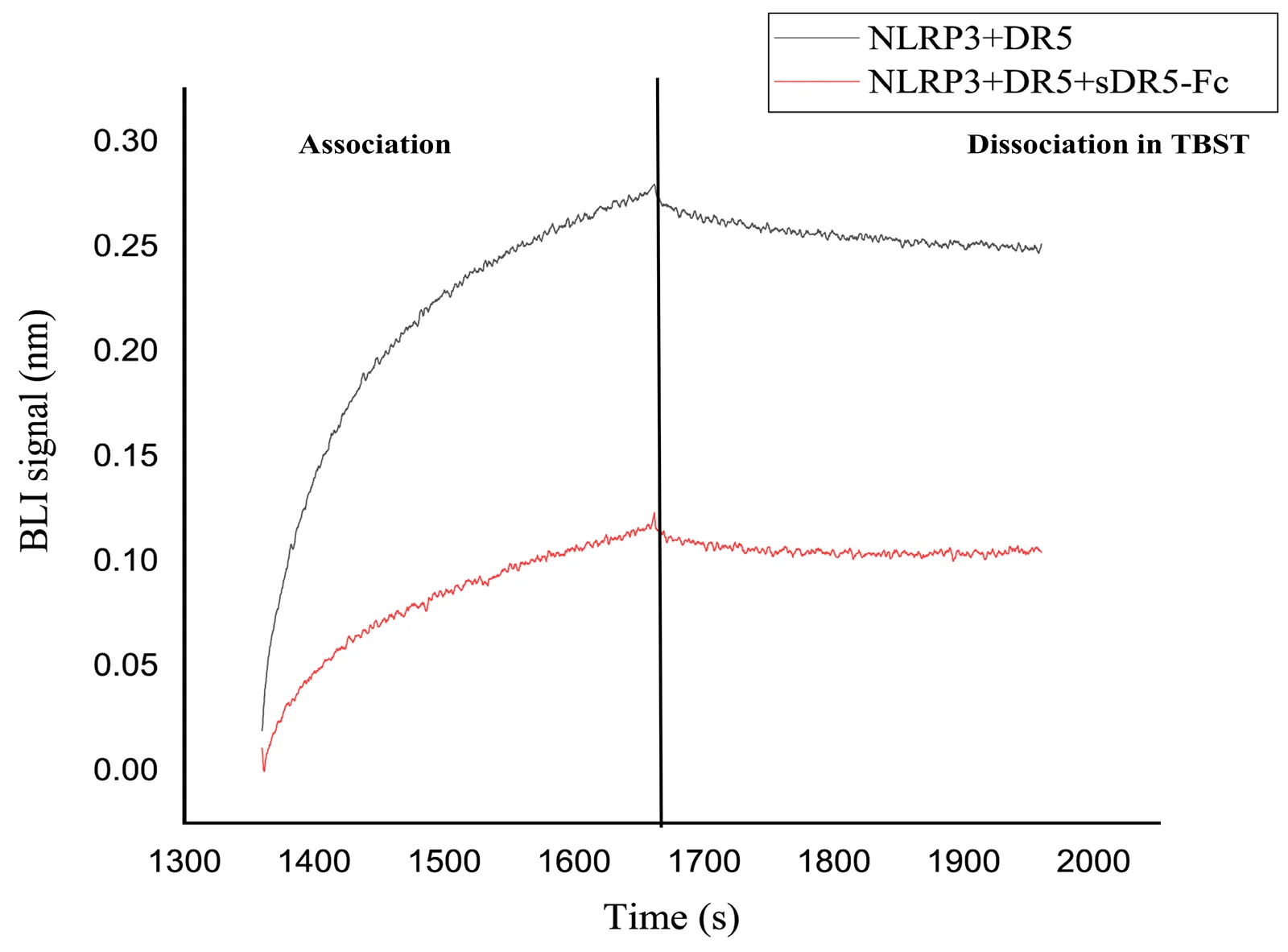
Biological layer interference technique.

The interaction between NLRP3 and DR5 was investigated using the biological layer interference technique to see if the introduction of sDR5-Fc may inhibit the binding of NLRP3 and DR5. Consistent with docking prediction, BLI data showed that sDR5-Fc could interfere with the recombinant-protein-level association between DR5 and NLRP3 in vitro, hinting that sDR5-Fc may modulate NLRP3-dependent pyroptosis under radiation stress (Figure 8).

#### 2.2.3. sDR5-Fc Inhibited the NLRP3/Caspase-1/GSDMD Pyroptosis in IR-Induced Enterocytes

**Figure 9:**
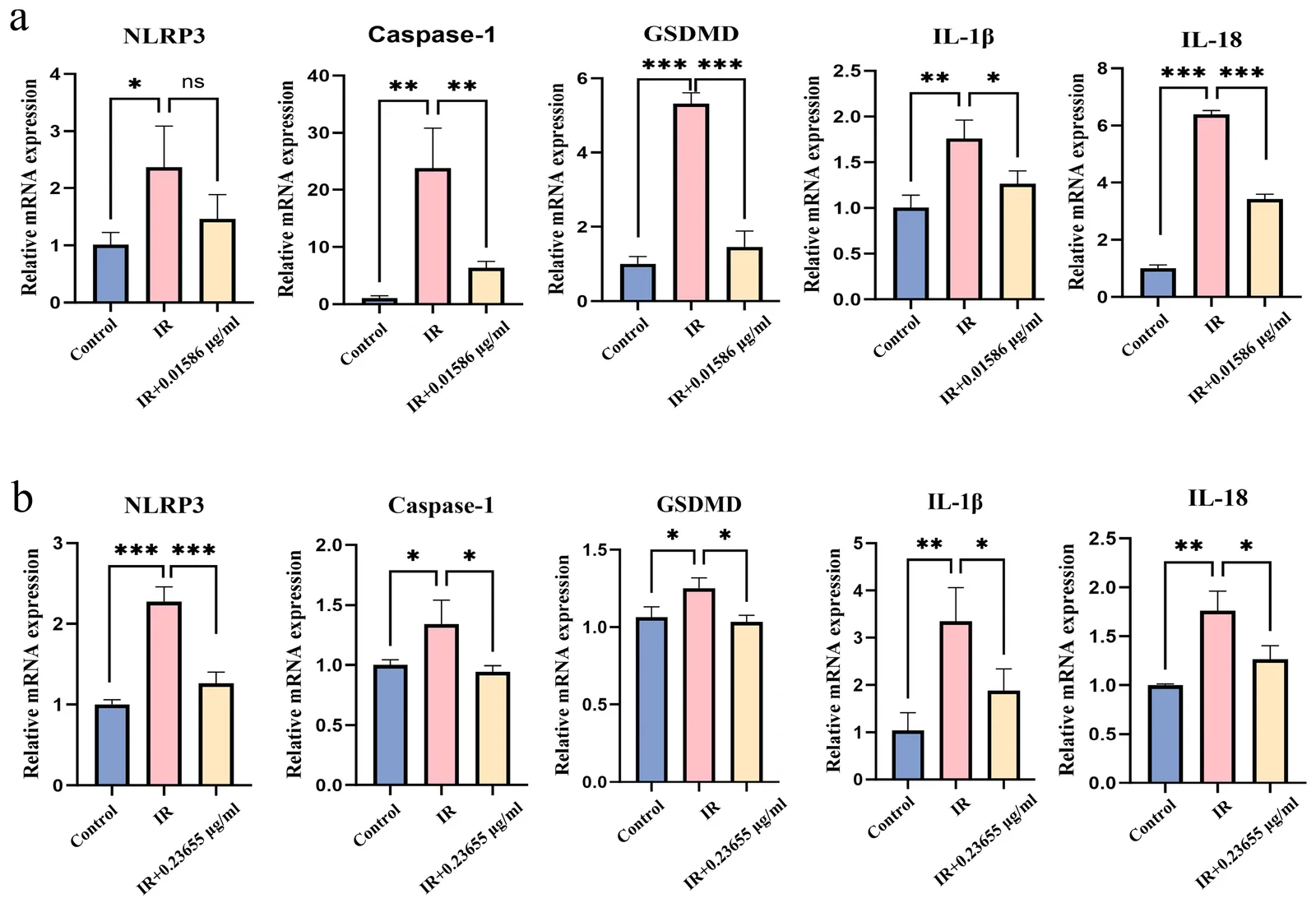
Effect of sDR5-Fc on genes related to pyroptosis in HIEC-6/IEC-6 cells after radiation exposure. (**a**) HIEC-6; (**b**) IEC-6. * *p* < 0.05, ** *p* < 0.01, *** *p* < 0.001 vs. IR group.

**Figure 10:**
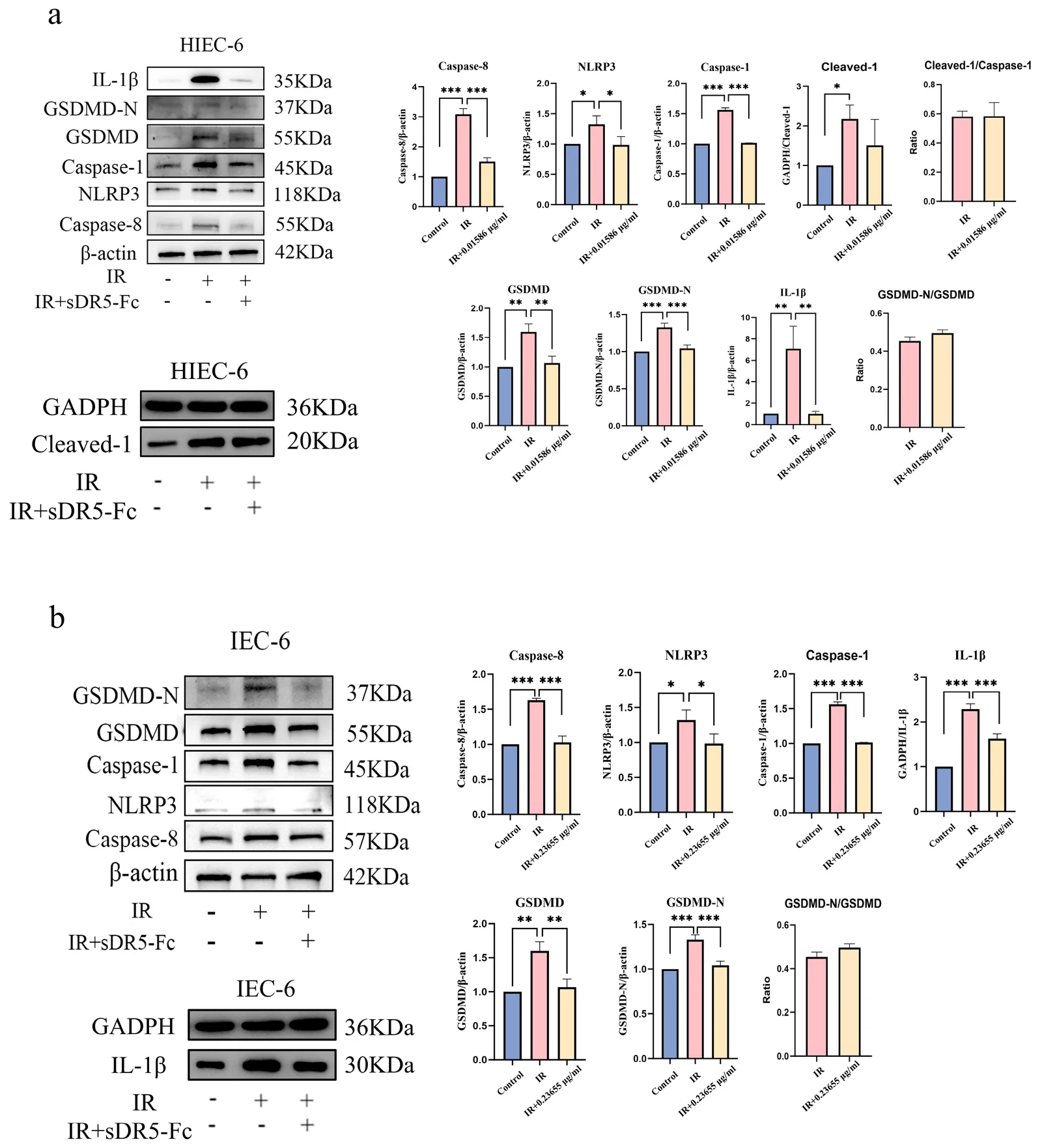
Effect of sDR5-Fc on proteins associated with pyroptosis in HIEC-6/IEC-6 cells after radiation exposure. (**a**) HIEC-6; (**b**) IEC-6. * *p* < 0.05, ** *p* < 0.01, *** *p* < 0.001 vs. IR group.

The NLRP3-mediated pyroptosis signaling pathway is a crucial inflammatory response mechanism. It has been observed that increased NLRP3 expression activates a series of downstream key molecules, including Caspase-1 and GSDMD. This results in the formation of pores in the cell membrane, increased cell permeability, membrane rupture, and ultimately cell pyrosis, which releases cell contents and inflammatory factors [60,61]. This study performed Western blotting and qRT-PCR to evaluate the effect of sDR5-Fc on NLRP3 inflammasome activation. qRT-PCR (Figure 9) revealed that irradiation significantly increased the expression of NLRP3, Caspase-1, GSDMD, IL-1β, and IL-18 mRNA in both cell lines. However, sDR5-F significantly reduced these levels compared with the IR group. Moreover, these data were validated by Western blot analysis (Figure 10), which revealed that the expression of Caspase-8, NLRP3, Caspase-1, Cleaved Caspase-1, GSDMD, GSDMD-N, and IL-1β was significantly increased in irradiated HIEC-6 and IEC-6 cells, whereas sDR5-Fc treatment significantly reduced these proteins. Western blot and qRT-PCR assays indicated that DR5 may activate the NLRP3/Caspase-1/GSDMD-mediated pyroptosis signaling pathway in irradiated enterocytes, while the DR5 antagonist, sDR5-Fc, significantly inhibits irradiation-induced pyroptosis.

### 2.3. In Vivo Studies

#### 2.3.1. sDR5-Fc Significantly Improved the Survival Time of 15 Gy γ-Irradiated Mice

**Figure 11:**
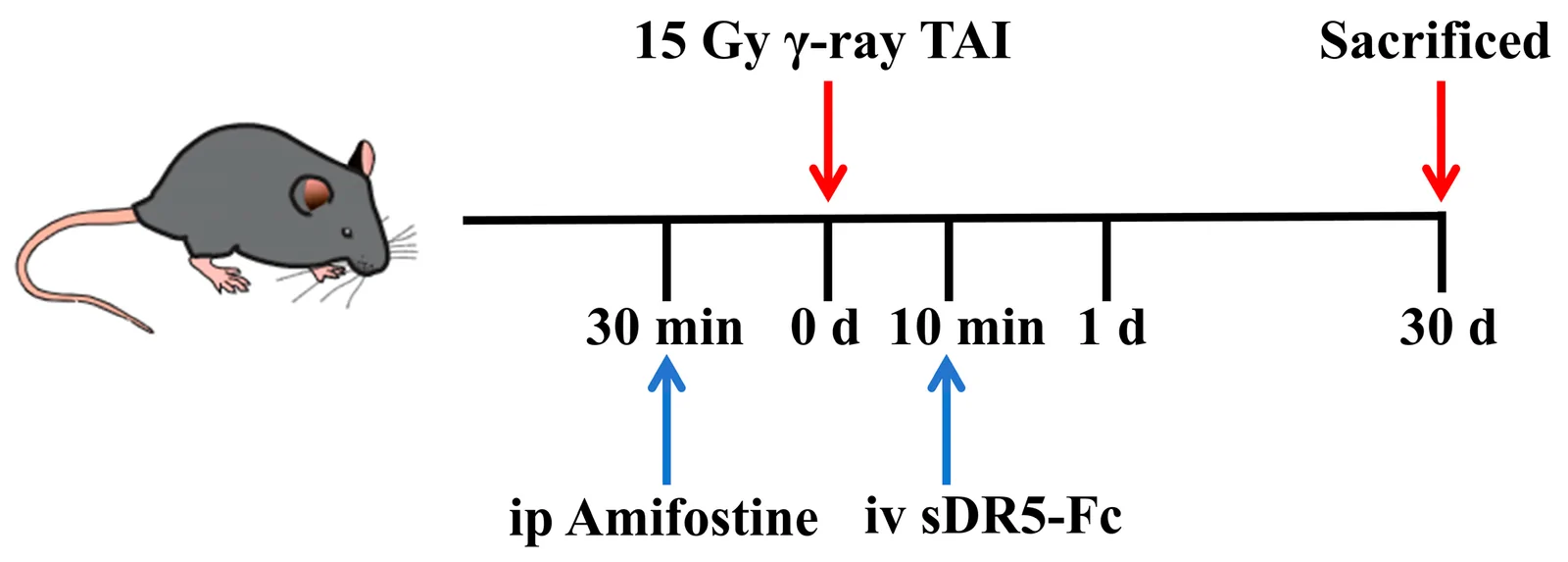
Schematic diagram of the experimental design of γ-ray-induced intestinal damage survival in mice.

**Figure 12:**
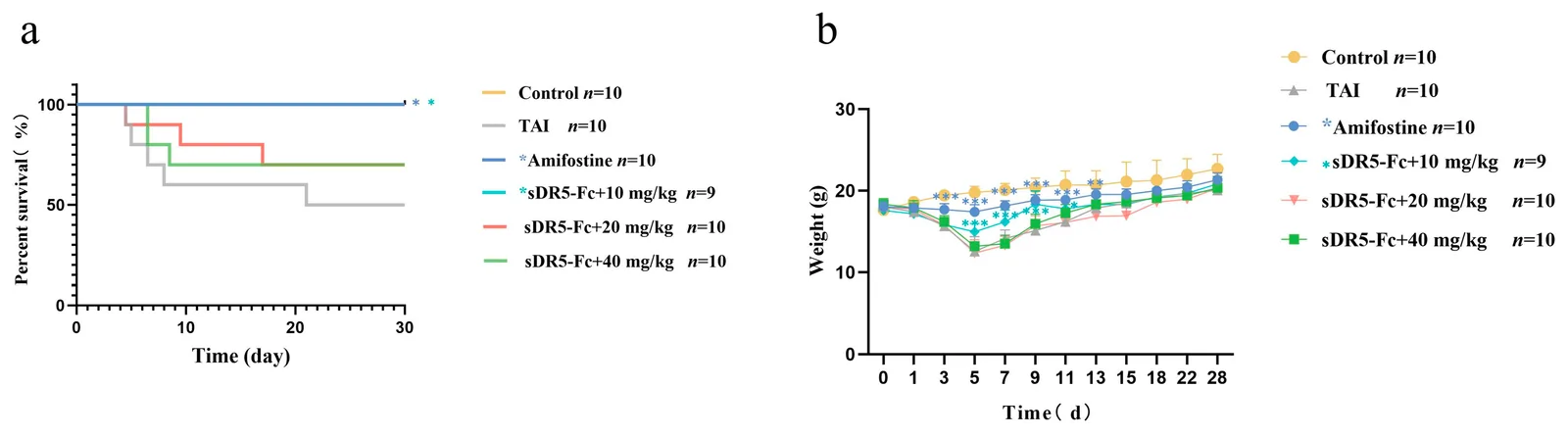
Effects of sDR5-Fc on survival and body weight in 15 Gy TAI mice. (**a**) Kaplan–Meier survival analysis of mice after 15 Gy TAI. (**b**) Body weight analysis of mice after 15 Gy TAI. * *p* < 0.05, ** *p* < 0.01, *** *p* < 0.001 vs. TAI group. (The 10 mg/kg sDR5 Fc group had 9 mice, which might be attributed to individual variances among the animals, as well as anesthetic errors during the experiment that led to individual mouse deaths).

The experimental design for the survival rate analysis is shown in Figure 11. The C57 BL/6 J mice were anesthetized using 1% pentobarbital sodium. Then, the total abdominal irradiation (TAI; single 15 Gy γ-radiation) was applied. The positive group (150 mg/kg amifostine) was injected intraperitoneally 15 min before irradiation, while different sDR5-Fc doses were injected immediately after irradiation into the tail vein. The survival rates and curves were shown in Figure 12a. The 30-day survival rate was 100% in both the positive group and the 10 mg/kg sDR5-Fc group (* *p* < 0.05 vs. TAI group), whereas the survival rates in the 20 and 40 mg/kg sDR5-Fc groups were 70%, which were still higher than the 50% survival rate of the irradiation group.

Figure 12b illustrates the body weight trajectories of various groups following irradiation. All groups had considerable weight loss by the 5th day post-irradiation, subsequently commencing a gradual recovery. The body weight of the 10 mg/kg sDR5-Fc group and the amifostine group was substantially greater than that of the irradiation group on days 5 to 11 and 3 to 13, respectively (* *p* < 0.05, ** *p* < 0.01, *** *p* < 0.001 vs. TAI group).

#### 2.3.2. sDR5-Fc Promoted the Blood Recovery of Irradiated Mice

**Figure 13:**
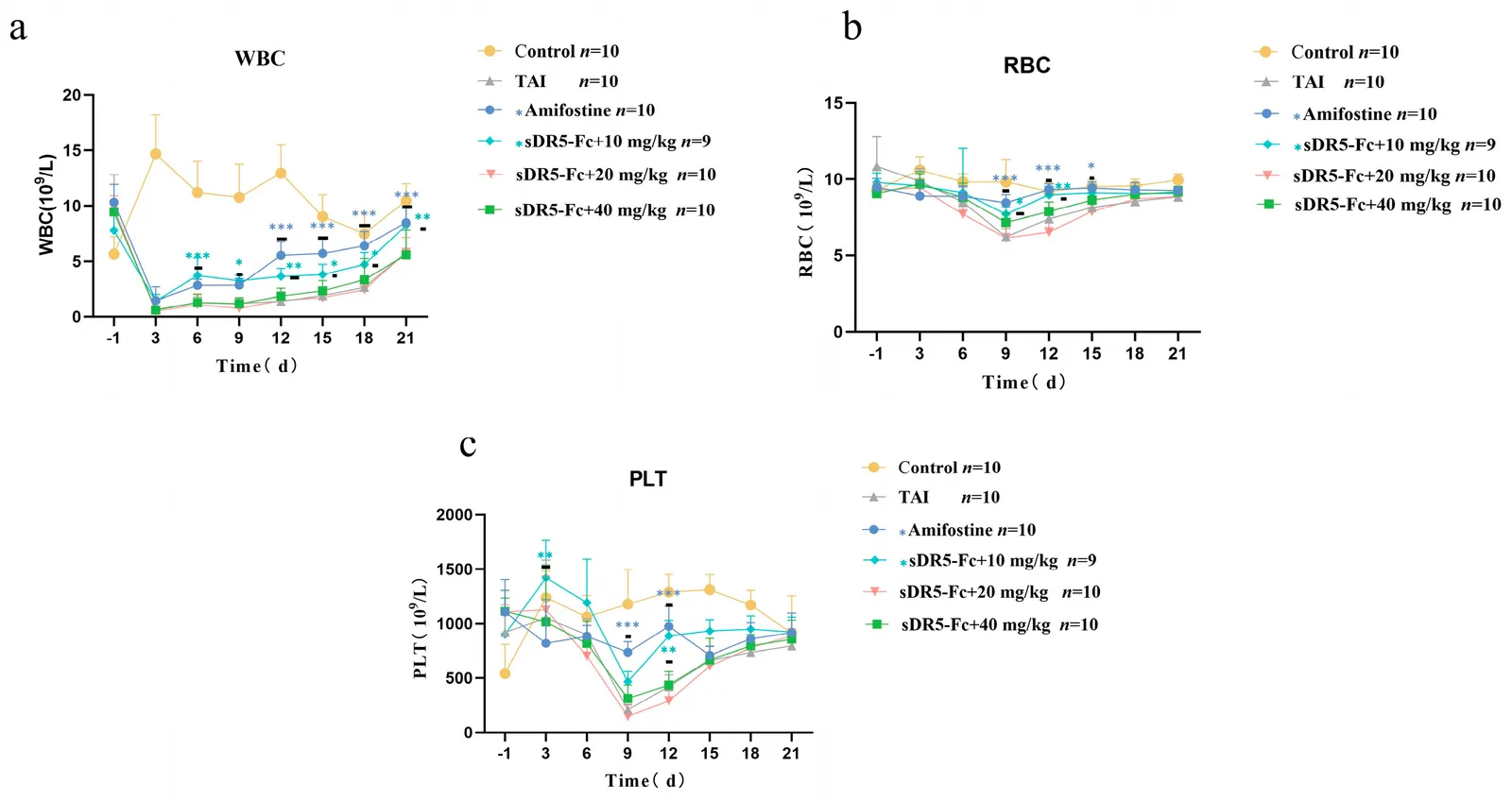
Effect of sDR5-Fc on the blood profile of 15 Gy TAI mice. (**a**) White blood cells (**b**), red blood cells, and (**c**) platelets of mice after 15 Gy TAI. * *p* < 0.05, ** *p* < 0.01, *** *p* < 0.001 vs. TAI group.

After a single high-dose irradiation, the levels of white blood cells (WBCs), red blood cells, and platelets in the peripheral blood of mice were substantially reduced (with the lowest levels occurring on days 3, 9, and 9 after irradiation, respectively), followed by a gradual recovery to normal levels by day 21 (Figure 13). Furthermore, sDR5-Fc administration significantly promoted the recovery of WBCs (Figure 13a). Moreover, from day 6 to day 21 after irradiation, the WBC levels were significantly higher in the 10 mg/kg sDR5-Fc group than those in the irradiated control group (* *p* < 0.05, ** *p* < 0.01, *** *p* < 0.001 vs. TAI group).

#### 2.3.3. sDR5-Fc Attenuated the Tissue Damage of Irradiated Mice

**Figure 14:**
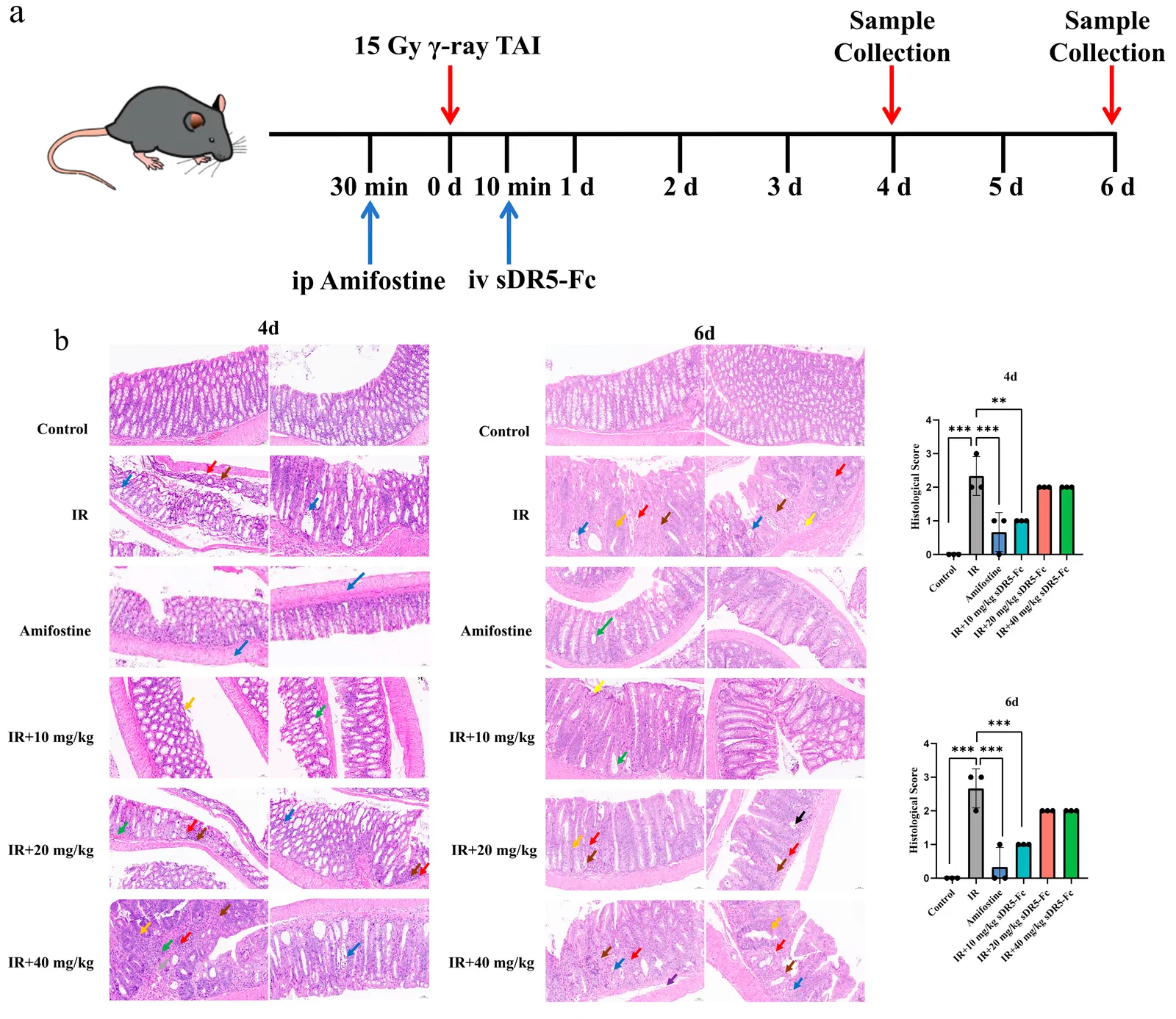

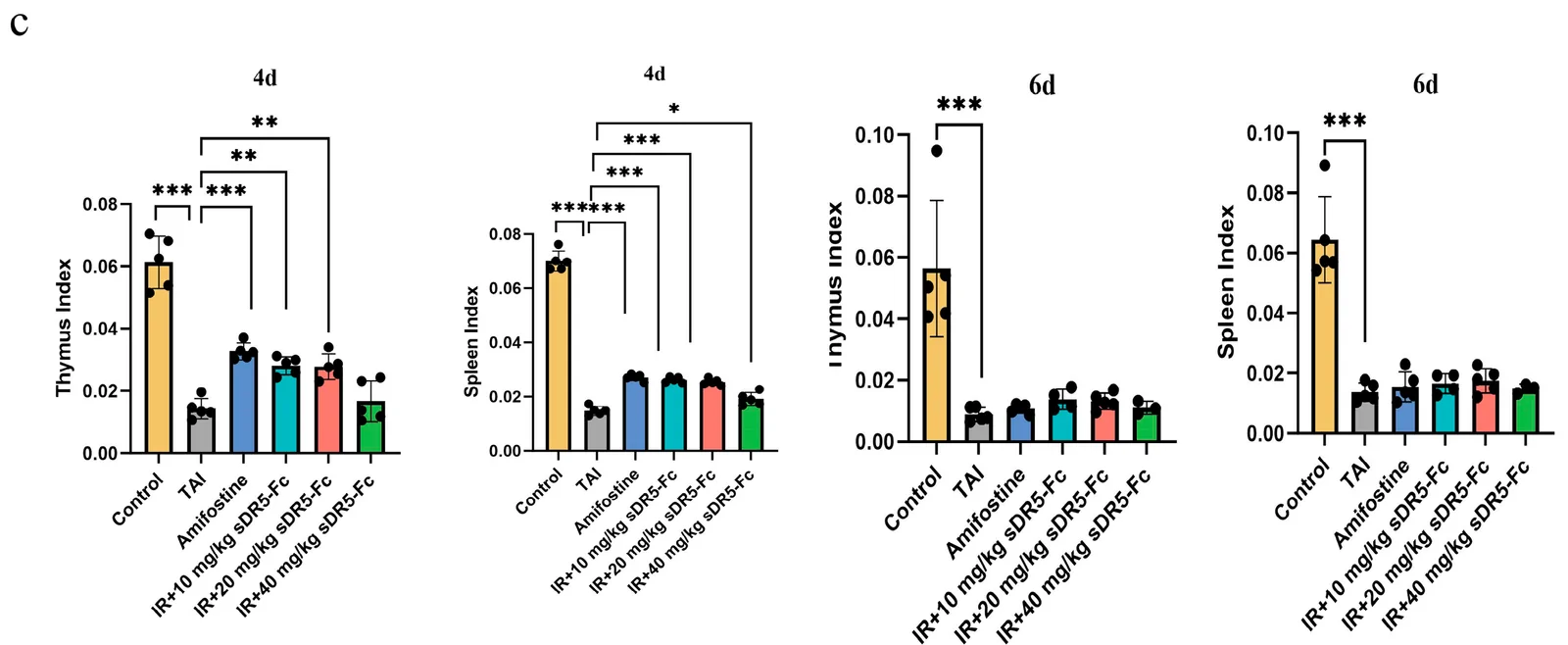
Effects of sDR5-Fc on radiation-induced tissue damage. (**a**) Schematic diagram of γ-ray-induced intestinal injury, sDR5-Fc administration, and sample collection in mice. (**b**) Representative images and H&E staining scores of colonic tissues in each group after 15 Gy TAI. (**c**) Organ indices of mice after 15 Gy TAI. * *p* < 0.05, ** *p* < 0.01, *** *p* < 0.001, vs. TAI group. Red arrow: lymphocytic infiltration. Blue arrows: increased dilation of intestinal glands in the lamina propria, flattened epithelial cells within the glands, and necrotic cellular debris present in the glandular lumen; there is also more cytoplasmic laxity of myocytes in the lamina propria. The yellow arrow: the damage reached the submucosa, where a slight lymphocytic infiltration is observed. Orange arrows indicate epithelial cell detachment and hyperplasia inside the mucosal layer, accompanied by observable nuclear fragmentation. Green arrows: sporadic vascular bruises in the lamina propria; many dilated intestinal glands with attenuated intestinal glandular epithelial cells; minimal lymphocytic and granulocytic infiltrates; Gray arrow: slight connective tissue hyperplasia in the submucosal layer; Black arrow: slight necrotic cellular debris; Purple arrow: myocyte cytoplasmic laxity and diminished staining; Dark blue arrow: a limited quantity of distended intestinal glands exhibiting flattened epithelial cells. The brown arrow indicates that the intestinal tissue exhibits numerous erosions, necrosis of mucosal epithelial cells, a decrease in the quantity of intestinal glands, and a small degree of fibrous connective tissue hyperplasia supplanting the original intestinal glands.

Figure 14a illustrates the pharmacological experimental design. To evaluate the protective efficacy of sDR5-Fc against radiation-induced colonic damage, colon tissue specimens from irradiated mice were analyzed using H&E staining (Figure 14b), and the spleen and thymus indices (Figure 14c) were computed at 4 and 6 days post-TAI, respectively. The standard histological alterations and their associated pathological scores revealed substantial pathological damage in the irradiation model group (*** *p* < 0.001 compared to normal control) (Figure 14b). Following treatment with various doses of sDR5-Fc or the positive control amifostine, colonic tissue damage indicated differing levels of recovery, particularly in the 10 mg/kg sDR5-Fc cohort, whereas amifostine significantly alleviated intestinal radiation injury (** *p* < 0.01, *** *p* < 0.001 compared to TAI control).

The spleen and thymus indices at 4 and 6 days post-irradiation were calculated (Figure 14c), and the results revealed that the indices were significantly decreased (* *p* < 0.05, ** *p* < 0.01, *** *p* < 0.001, vs. normal control), suggesting significant functional damage caused by irradiation. Whereas different doses of sDR5-Fc or amifostine increased these indices to varying degrees, particularly on the 4th day post-irradiation, which was statistically significant (* *p* < 0.05, ** *p* < 0.01, *** *p* < 0.001, vs. TAI group).

#### 2.3.4. sDR5-Fc Significantly Reduced the Inflammatory Response of Irradiated Mice

**Figure 15:**
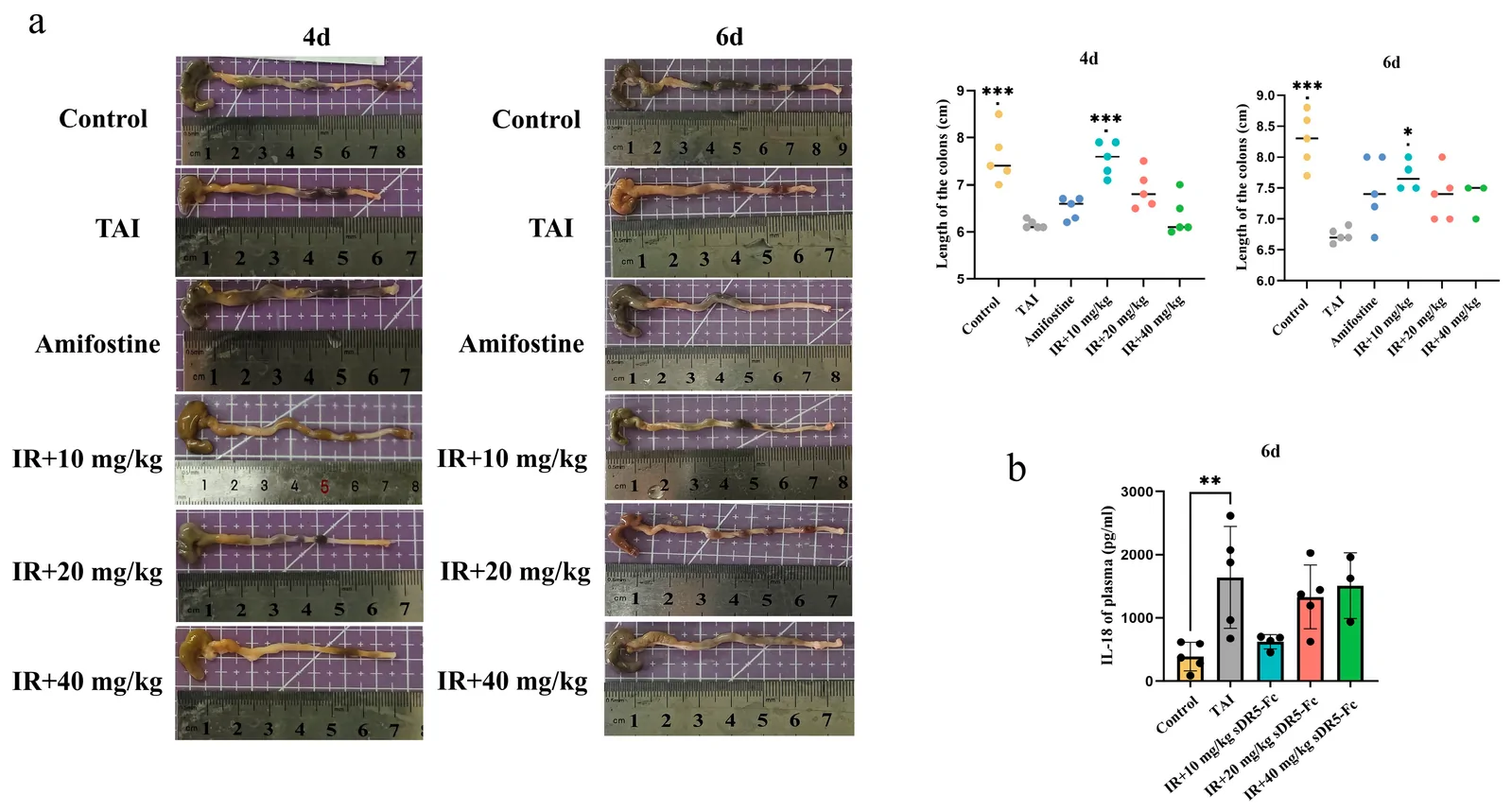
Effect of sDR5-Fc on the inflammatory response induced in mice after 15 Gy TAI. (**a**) Representative images of the colon of each group of mice after 15 Gy TAI and quantification of colon length. (**b**) Plasma levels of IL-18 in mice. * *p* < 0.05, ** *p* < 0.01, *** *p* < 0.001 vs. TAI group.

It has been confirmed that colon length shortening is a significant manifestation of intestinal inflammation triggered by localized abdominal irradiation [47]. IL-18, an inflammatory cytokine, serves as an essential marker of inflammatory reactions and is especially effective in confirming the presence of pyroptosis [62,63,64]. Therefore, this study assessed the colon length and plasma IL-18 levels to determine the potential effects of sDR5-Fc on mitigating radiation-induced inflammatory responses in vivo. It was found that the colon length was significantly decreased on days 4 and 6 after irradiation, respectively (*** *p* < 0.001 vs. normal control) (Figure 15a); however, the administration of different doses of sDR5-Fc or amifostine increased the colon length to varying degrees, mainly in the 10 mg/kg sDR5-Fc group, and the difference was statistically significant (* *p* < 0.05, *** *p* < 0.001 vs. TAI group). Further, the level of the inflammatory factor IL-18 was substantially higher in the irradiation mice group (** *p* < 0.01 vs. normal control), while sDR5-Fc reduced its level to varying degrees (Figure 15b).

#### 2.3.5. sDR5-Fc Inhibited Irradiation-Induced Pyroptosis in Mice

**Figure 16:**
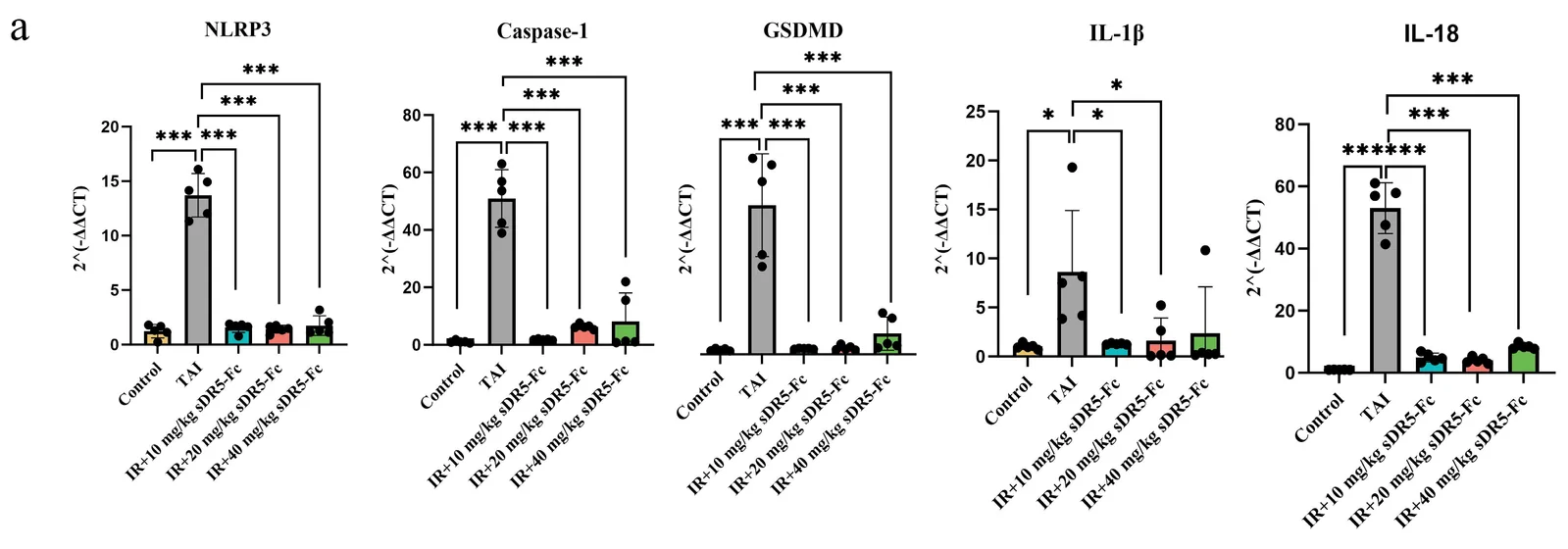

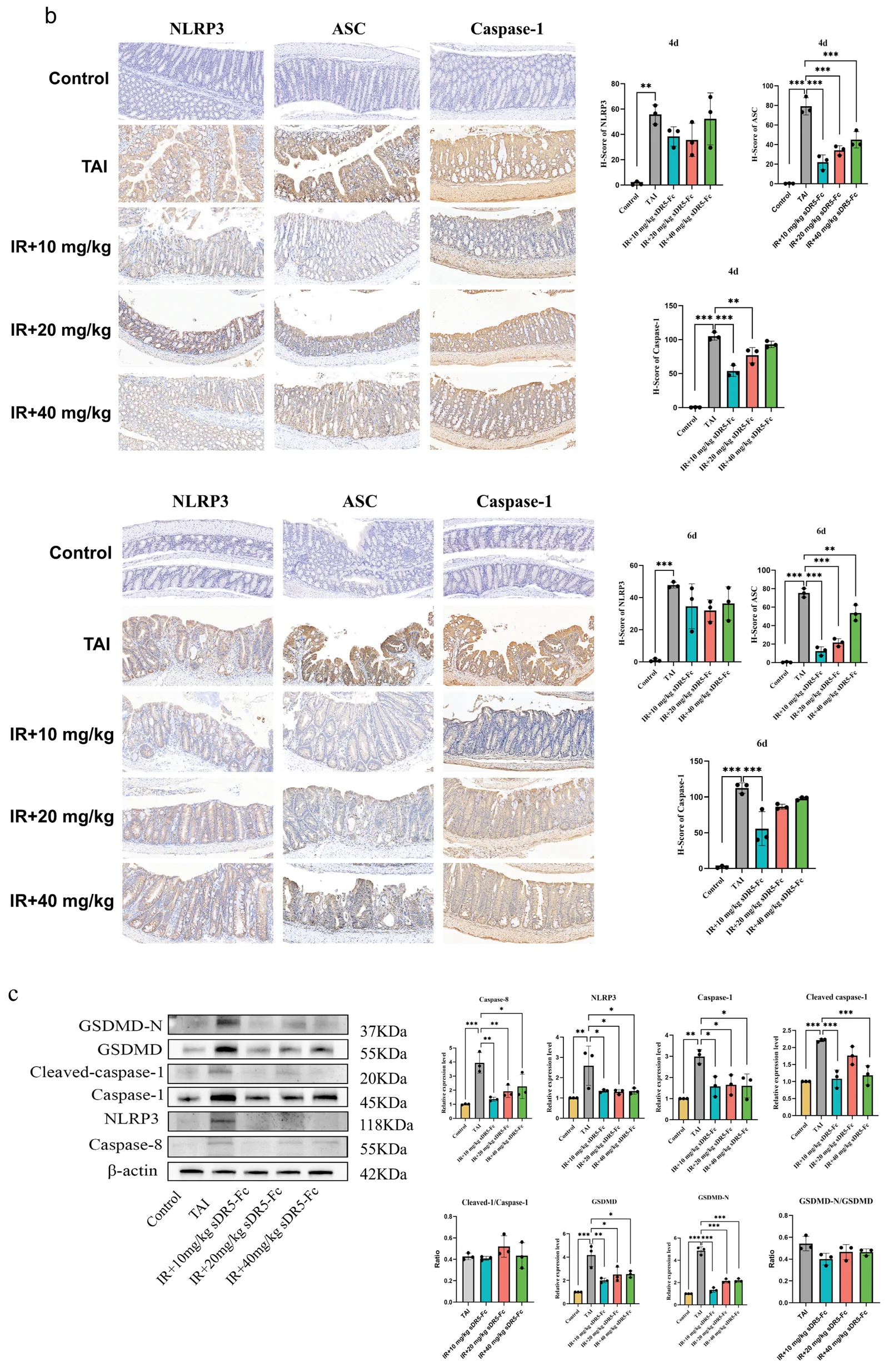
Effect of sDR5-Fc on focal death in mice after 15 Gy TAI. (**a**) qRT-PCR detection of pyroptosis-related gene expression in the small intestine after 15 Gy of TAI. (**b**) Immunohistochemical and (**c**) Western blot analyses of pyroptosis-associated proteins in the colon after 15 Gy TAI. * *p* < 0.05, ** *p* < 0.01, *** *p* < 0.001 vs. TAI group.

The classical NLRP3-mediated pyroptosis signaling pathway was investigated in intestinal tissues of mice after irradiation and sDR5-Fc administration. The qRT-PCR (Figure 16a) revealed that the levels of NLRP3, Caspase-1, GSDMD, IL-1β, and IL-18 were significantly increased in the small intestine after irradiation (* *p* < 0.05, *** *p* < 0.001 vs. normal control). However, different doses of sDR5-Fc increased Caspase-1 activity, while decreasing the NLRP3, Caspase-1, GSDMD, IL-1 β, and IL-18 expression (* *p* < 0.05, *** *p* < 0.001 vs. TAI control). Similarly, immunohistochemistry analysis (Figure 16b) indicated that irradiation increased NLRP3, Caspase-1, and ASC levels in colon tissue, while administration of different doses of sDR5-Fc alleviated these changes. Western blot analysis (Figure 16c) showed that Caspase-8, NLRP3, Caspase-1, Cleaved Caspase-1, GSDMD, and GSDMD-N protein levels were significantly elevated in the irradiated mice (** *p* < 0.01, *** *p* < 0.001 vs. normal control) while significantly decreased after treatment with different doses of sDR5-Fc (* *p* < 0.05, ** *p* < 0.01, *** *p* < 0.001 vs. TAI control). These results indicate that a single 15 Gy TAI induces significant intestinal pyroptosis and that the DR5 antagonist, sDR5-Fc, can effectively inhibit pyroptosis and mitigate radiation-induced enteric injury.

## 3. Discussion

Pyroptosis is a type of programmed cell death that is associated with inflammatory responses. It has been identified in various cell types, including enterocytes, smooth muscle cells, and cardiomyocytes, and is related to the pathogenesis of several diseases [65,66]. The literature suggests that pyroptosis of intestinal cells is significantly associated with the development of several intestinal diseases. Pyroptosis in intestinal cells is common in the intestinal tract, caused mainly by alterations in the intestinal environment that induce cell death. While the precise mechanism of intestinal cell death is not fully understood, researchers have progressively clarified certain aspects using experimental animal and cellular models. Chronic intestinal inflammation [67], disruption of cell membranes [68], and lack of intracellular calcium ions [69] may significantly promote intestinal cellular pyroptosis. Radiation enteritis, which is mainly caused by ionizing radiation damage to the intestine, is a common complication after radiotherapy in patients with pelvic or abdominal tumors. Furthermore, it has been observed that low-dose 4 Gy whole-body irradiation does not significantly damage the intestinal barrier but rapidly induces significant cellular pyroptosis in enterocytes [70]. Cellular pyroptosis can cause severe disturbances in the homeostasis of the enteric environment by stimulating a cascade of pathologies and inflammation [71]. Mechanistically, enteric cellular pyroptosis disrupts the distal gut microenvironment through sustained release of pro-inflammatory cytokines and damage-associated molecular patterns, which drives distal mucosal barrier hyperpermeability and microbial dysbiosis. In comparison with the pyroptosis-driven barrier disruption observed in our study, previous studies have validated multiple strategies for mucosal barrier stabilization: Li et al. and Chen et al. demonstrated that microbial modulation and polysaccharide interventions can reinforce intestinal barrier integrity and maintain mucosal homeostasis [72,73,74]. Furthermore, Li et al. detailed targeted mucosal homeostasis architectures that offer precision therapeutic paradigms for preserving gut barrier function under inflammatory conditions [75].

As demonstrated previously [27], sDR5-Fc acts as a soluble decoy receptor to neutralize autocrine/paracrine membrane-bound TRAIL, preventing TRAIL-DR5 binding and DISC formation at the cell surface. In the present study, we did not conduct experiments to evaluate DR5 receptor endocytosis; our data support a model in which sDR5-Fc interrupts upstream TRAIL-DR5 signaling via ligand neutralization, rather than by altering DR5 internalization.

The NLRP3 inflammasome-dependent release of IL-1β and IL-18 propagates secondary inflammatory cascades within the irradiated intestinal microenvironment, driving macrophage activation and tissue destruction. As described in recent work on intestinal inflammatory axis regulation and macrophage reprogramming [76], suppression of inflammasome signaling can redirect macrophage polarization toward a tissue-repair phenotype, alleviating radiation-triggered mucosal damage. Our findings indicate that sDR5-Fc-mediated inhibition of TRAIL-DR5 signaling attenuates this NLRP3-driven inflammatory cascade, which may contribute to reduced macrophage-mediated injury in the irradiated intestine.

DR5 is a type I transmembrane receptor protein that binds the ligand TRAIL through its external cysteine-rich domain (CRD), while its intracellular death domain transmits apoptosis signals [77,78]. TRAIL-DR5 interaction prompts DR5 trimerization, enabling the recruitment of Fas-associated death domain protein (FADD) and procaspase-8 to assemble the death-inducing signaling complex (DISC), thus beginning apoptosis. DR5 has low expression in normal tissues but high expression in various tumor cells; therefore, the TRAIL-DR5-mediated apoptotic signaling pathway is crucial for tumor suppression and immunological modulation. Thus, DR5 agonists are promising candidates for targeted cancer therapy [79].

Recent advancements in understanding the regulatory mechanisms of DR5 have broadened its therapeutic significance beyond oncology, highlighting the potential of DR5 antagonists in apoptosis-related disorders [80,81]. sDR5-Fc, a recombinant fusion protein consisting of the human soluble extracellular DR5 domain (sDR5) and the Fc region, functions as an optimal DR5 antagonist. It maintains a strong affinity for TRAIL but is incapable of transmitting downstream apoptotic signals, therefore competitively obstructing TRAIL/DR5-mediated apoptosis [41,82,83]. Recently, several studies have demonstrated that DR5 is significantly up-regulated in target organs under stress-associated pathological conditions, such as cerebral ischemia–reperfusion injury [33,34] and acute severe hepatitis [84]. This upregulation induces excessive apoptosis of functioning cells, exacerbating disease progression and tissue damage. The administration of the DR5 antagonist sDR5-Fc efficiently alleviates target organ dysfunction and facilitates tissue healing in these conditions. Our previous research [27] showed pathogenic overexpression of DR5 in the thymus and spleen of mice with severe hematopoietic acute radiation syndrome. Furthermore, sDR5-Fc therapy substantially reduced radiation-induced organ damage and increased survival rates, highlighting its therapeutic efficacy in severe acute radiation injury.

The mechanisms underlying radiation-induced cell death are complex and multifaceted. In addition to excessive apoptosis, the overactivation of other cell death regulatory mechanisms, including pyroptosis [85] and ferroptosis [86], etc., may also influence the biological effects of radiation exposure. Caspase-8, a crucial downstream effector of the TRAIL/DR5 signaling system, has been shown to trigger pyroptosis by cleaving GSDMD into its active form, indicating the potential of DR5 in regulating the pyroptosis signaling pathway [87,88,89].

This study investigated whether the DR5 antagonist, sDR5-Fc, can alleviate DR5-mediated pyroptosis-related intestinal radiation damage and explore its underlying mechanisms. Furthermore, a radiation-induced intestinal epithelial cell (HIEC-6 and IEC-6) injury model was established using a single high-dose (15 Gy) γ-ray irradiation. Then, the morphological changes associated with pyroptosis were analyzed using SEM. Moreover, key biochemical markers of pyroptosis (LDH release), potassium ion efflux, and oxidative stress levels (ROS, MDA, and SOD) were evaluated. The mRNA and protein expression of pyroptosis-related molecules (NLRP3, Caspase-1, GSDMD-N, IL-1β, and IL-18) were also evaluated. All results revealed that high-dose irradiation induced considerable oxidative stress and pyroptosis in enterocytes, whereas sDR5-Fc therapy significantly reduced markers associated with pyroptosis-related damage. Subsequently, in vivo assays were conducted to validate the protective effects of sDR5-Fc on mice suffering from radiation-induced enteritis after exposure to high-dose (15 Gy) γ-ray TAI. Notably, in this study, sDR5-Fc exhibited a non-monotonic dose–response relationship in vivo. The maximum radiation protection effect appeared at 10 mg/kg, while higher doses (20 mg/kg and 40 mg/kg) actually reduced therapeutic efficacy. Many protein-based drugs, cytokines, and fusion proteins exhibit inverted U-shaped non-monotonic dose effects, which may be due to target receptor saturation at high concentrations, non-targeted immune interference caused by Fc fragments, or compensatory pro-inflammatory feedback from overdose drug exposure. To fully understand this non-monotonic behavior, further pharmacodynamic studies are needed, so under current experimental conditions, 10 mg/kg is defined as the optimal therapeutic dose. These studies further substantiated that sDR5-Fc significantly mitigated pyroptosis in intestinal and colonic tissues, facilitated the recovery of peripheral blood cells, including leukocytes, erythrocytes, and platelets, enhanced spleen and thymus organ indices, and elevated the 30-day survival rate of the irradiated mice. In contrast to the positive control, amifostine, which must be delivered prophylactically within 30 min before radiation exposure [90,91], the efficacy of sDR5-Fc when given post-radiation underscores its considerable potential and benefits for emergency applications.

Moreover, to investigate the potential molecular mechanism by which sDR5-Fc inhibits pyroptosis, molecular docking analysis was carried out between DR5 and NLRP3, a key pattern recognition receptor (PRR) in the pyroptosis signaling pathway. The results revealed a strong binding affinity between the two molecules. Furthermore, the molecular interaction assays validated the robust, direct binding between DR5 and NLRP3 in vitro, which was significantly inhibited by sDR5-Fc. Furthermore, both human- and murine-derived intestinal epithelial cell lines (HIEC-6 and IEC-6) showed that radiation exposure substantially upregulated mRNA and protein expression of key pyroptosis pathway-related molecules, including NLRP3, Caspase-1, GSDMD, IL-1β, and IL-18. Whereas sDR5-Fc intervention effectively inhibited these molecules at both transcriptional and translational levels. Collectively, our in silico and recombinant-protein assays support a potential interplay between DR5 and NLRP3 that may contribute to inflammasome activation upon irradiation, which then stimulates downstream signaling events, including Caspase-1 and GSDMD cleavage, leading to cell membrane damage and the release of inflammatory mediators such as IL-1β and IL-18, ultimately resulting in pyroptosis. sDR5-Fc inhibits activation of the NLRP3/Caspase-1/GSDMD signaling pathway by competitively binding to DR5, suppressing the excessive pyroptosis induced by radiation.

## 4. Materials and Methods

### 4.1. Animals

Healthy male C57BL/6J (17–22 g) mice were procured from the Viton Lihua Animal Breeding Center (Beijing, China) and housed in the Laboratory Animal Center barrier feeding system in standard individually ventilated cages at a 12 h light/dark cycle, at 18–25 °C, and in 50–60% humidity with ad libitum access to food and water. All in vivo analyses were approved by the National Institutes of Health Guide for the Care and Use of Laboratory Animals and followed the guidelines of the Animal Care and Welfare Committee of the Beijing Institute of Radiological Medicine (No. IACUC-DWZX-2024-P626, 4 March 2024).

In the survival study, after acclimatization for 3–7 days, the body weight of each mouse was measured by an electronic balance. Block randomization was performed based on body weight to allocate animals into different experimental groups, with 10 mice in each group. Briefly, all mice were sorted in ascending order according to body weight. Mice with similar body weight were assigned to one block. Within each block, animals were randomly distributed to each experimental group using a random number table. The baseline body weight of mice among groups was compared to confirm no significant inter-group difference. Each experimental group was initially assigned ten mice. In the 10 mg/kg sDR5-Fc group, one mouse died before irradiation due to an anesthesia-related complication. A necropsy showed no organ lesions related to the test substance, confirming that the death was a one-off event related to anesthesia, not to the pharmacological or toxic effects of the test substance. According to our pre-approved IACUC protocol and pre-defined exclusion criteria, this animal was excluded from all subsequent data collection and statistical analysis. Therefore, the final sample size for the 10 mg/kg sDR5-Fc group was *n* = 9, while the other groups remained at *n* = 10. This mouse died before specimen collection, and no valid experimental indicators were obtained, so its data was directly excluded without artificial selective removal of experimental data. This loss of one sample did not significantly affect the overall conclusions of the study. All animal experiments were carried out strictly following the guidelines of the Animal Care and Welfare Committee.

### 4.2. Cell Culture

Human small intestinal mucosal epithelial cells (HIEC-6) and Intestinal Epithelioid Cell (IEC-6) were cultured in Dulbecco’s Modified Eagle Medium (812619, Gibco, Waltham, MA, USA) augmented with 10% fetal bovine serum (SV30208.02, Cytiva HyClone, Logan, UT, USA) and 1% Penicillin–Streptomycin (15140-122, Gibco, USA) at 37 °C in 5% CO_2_.

### 4.3. γ-Ray Radiation

Mice were locally irradiated by exposure to the ^60^Co radiation source at a dose rate of 173.35 R/min and a radiation dose of 15 Gy. For the in vitro assay, both cell lines were exposed to a 15 Gy radiation dose at 170.14 R/min.

### 4.4. Scanning Electron Microscope (SEM) Assay

Briefly, HIEC-6/IEC-6 cells were irradiated with 15 Gy γ, immediately treated with sDR5-Fc, and cultured for 24 h. The cells were then collected and subjected to “fixation–post-fixation–dehydration–drying–conductive treatment of samples”. The samples were observed under SEM and then imaged.

### 4.5. Lactate Dehydrogenase (LDH) Assay

The culture supernatant was transferred to an EP tube and centrifuged at 2000 rpm for 5 min to collect supernatant for the LDH activity assay based on the protocol of the assay kit (Nanjing Jiancheng Bioengineering Institute, Nanjing, China). Briefly, the samples were mixed with matrix buffer and coenzyme I at 37 °C for 15 min, followed by treatment with 2,4-dinitrophenylhydrazine at 37 °C for 15 min, and then sodium hydroxide solution at room temperature for 5 min. Then, the absorbance was detected at 440 nm.

### 4.6. Flow Cytometry Determination

Both cell lines were subjected to 15 Gy γ irradiation and immediately treated with sDR5-Fc. After 6 and 24 h of incubation, the cells were collected. Furthermore, 1 unit of FAM-YVAD-FMK (655, Immunochemistry, Bloomington, MN, USA) was dissolved in 50 μL of DMSO to prepare a reserve solution. Then, 1 unit of the reserve solution was diluted (1:5 ratio) with PBS to make the working solution; 10 of which was added to 290 μL cell suspension at 37 °C in the dark for 1 h, centrifuged, and resuspended on ice with 300 μL wash buffer. Samples were then mixed with 1.5 μL PI staining solution in a dark room for 5 min, washed, and resuspended with 300 μL wash buffer. For flow cytometry detection, the FLICA excitation wavelength was 488–492 nm, and the emission wavelength was 515–535 nm, whereas for PI, the excitation wavelength was 488–492 nm, and the emission wavelength was 610–717 nm.

### 4.7. Immunofluorescence Staining

Both cell lines were subjected to 15 Gy γ irradiation and immediately treated with sDR5-Fc. After 6 and 24 h of incubation, the cells were collected and fixed in 4% paraformaldehyde for 20 min. After removing paraformaldehyde, cells were circled and sealed. The sealing solution in the circle was then removed, and PBS was added dropwise; the primary antibodies Caspase-1 (ET1608-69, Huaan Biological, Hangzhou, China) and GSDMD-N (HA7211144, Huaan Biological) were added in a certain proportion and smeared flat in the wet box at 4 °C overnight. Then, the cells were washed thrice, each time for 5 min, dried slightly, and covered with a drop of secondary antibody (GB21303, Servicebio, Wuhan, China) corresponding to the primary antibody in the histochemical kit in the circle for 50 min at room temperature. Sections were washed in PBS (pH 7.4) on a decolorizing shaker, shaking 3 times for 5 min each time, followed by gentle shaking to dry and treatment with DAPI staining solution for 10 min at room temperature in the dark. The smears were placed in PBS (pH 7.4) on a decolorizing shaker and washed by shaking 3 times for 5 min each time, followed by gentle shaking to dry. Subsequently, the samples were sealed with an anti-fluorescence quenching sealer, observed under a fluorescence microscope, and imaged. For DAPI (blue light), the UV excitation wavelength was 330–380 nm, and the emission wavelength was 420 nm. For FITC (green light), the excitation wavelength was 465–495 nm, and the emission wavelength was 515–555 nm. For CY3 (red light), the excitation wavelength was 510–560 nm, and the emission wavelength was 590 nm.

### 4.8. Detection of Extracellular K^+^ Efflux by ICP-MS

Both cell lines were subjected to 15 Gy γ irradiation and immediately treated with sDR5-Fc. After 24 h, cell supernatants were collected to assess the potassium standard solution profile and quality control of samples for nitrification. The ICP-MS parameters were as follows: RF power = 1150 W, RF matching = 1.8 V, sampling depth = 10 mm, concentric nebulizer, plasma flow rate = 15 L/min, acquisition mode = TRA, Omega deflection voltage = 105 V, Omega lens voltage = 13.6 V, and integration time = 1.0 s. Potassium ion concentration was detected.

### 4.9. Molecular Docking of NLRP3 with DR5 Protein

Molecular docking analysis was carried out to assess the rigid protein-protein docking relationship between NLRP3 and DR5 using GRAMM-X (http://gramm.compbio.ku.edu/, accessed on 12 May 2025). The protein structural domains of NLRP3 and DR5 were obtained from the Protein Data Bank (PDB) database (http://www.rcsb.org/, accessed on 12 May 2025). PyMOL (version 2.4) and PDBePISA (https://www.ebi.ac.uk/pdbe/pisa/, accessed on 12 May 2025) were employed for protein–protein interactions and further visual analyses.

### 4.10. Bio-Layer Interference Analysis

Molecular interactions were used to study the interaction between NLRP3 (HY--P701027, MCE) and DR5 (RPA818Hu02, Cloud-Clone Corp, Wuhan, China). Furthermore, a 10 μg/mL working solution of NLRP3 protein, a 2000 nM and a 1000 nM working solution of DR5 protein, and a 2000 nM working solution of sDR5-Fc protein were prepared. These three working solutions were then added to the corresponding 96-well plates. The anti-GST (18-5096; Sartorius, Göttingen, Germany) biosensor was activated with PBST, which was also used as a running buffer. The test parameters were set as follows: an oscillation rate of 1000 rpm and a stable plate temperature of 30 °C. The GST biosensor was initially attached to NLRP3 for baseline measurements (180 s), bonded to DR5 for 200 s, and then dissociated in buffer for 300 s. Analyses were conducted with Octet BLI Analysis Software version 12.2.

### 4.11. Western Blot

HIEC-6 and IEC-6 cell supernatants were discarded, cells were washed twice with PBS, and then treated with RIPA lysate (Cat. No.: WB3100, New Saimei Bio, Suzhou, China) containing 1 mM PMSF (GRF101, Yatase, Xi’an, China). Furthermore, colon tissue at 80 °C was placed on ice, then melted to room temperature, cut into small pieces with scissors, weighed 0.1–0.2 g, and mixed with grinding beads in 1 mL of RIPA lysate (RIPA:PMSF = 100:1). The tissue was ground until no tissue clumps remained. Subsequently, the BCA concentration assay was performed (Cat. No.: WB6501, New Saimei Bio). Briefly, after adjusting the concentration, proteins were treated with 5× sample buffer (LT101, Yatase), denatured at a high temperature for 10 min, and subjected to electrophoresis. Then, the proteins were transferred to membranes, blocked using skimmed milk (P1622, Beijing Applygen Gene Technology Co., Ltd., Beijing, China), and treated with primary antibodies (β-actin (C1313, Applygen Techologies Inc., Beijing, China), NLRP3 (ab263899, Abcam), Caspase-1 (ab179515, Abcam, Cambridge, UK), Cleaved Caspase-1 (4199, CST), GSDMD (ab219800, Abcam) 1:1000) at 4 °C overnight. The proteins were then washed with TBST (B1009, Applygen Techologies Inc., Beijing, China) for 10 min × 3 times, and then probed with secondary antibody (C1308-1, Applygen Techologies Inc., Beijing, China; C1309-1, Applygen Techologies Inc., Beijing, China) (1:5000) at room temperature for 1.5 h, washed with TBST for 10 min thrice, and reacted with ECL luminescent solution (P10200, New Saimei Bio, Suzhou, China) to develop bands, which were analyzed by ImageJ 1.53a software.

### 4.12. RT-qPCR

The NLRP3, Caspase-1, GSDMD, IL-1β, IL-18, and β-actin primer sequences were designed using the NCBI GeneBank database for human, rat, and mouse and synthesized by Sangon Bioengineering (Shanghai, China) Co., Ltd. (Table 1, Table 2 and Table 3). The relative mRNA expression was calculated using the 2^−ΔΔCT^ method for three biological replicates; 3 replicate wells/sample. β-actin was used as the internal reference.

### 4.13. Cellular RNA Extraction

HIEC-6 and IEC-6 cell supernatant was discarded, cells were washed twice with PBS, and then treated with 500 μL FreeZol Reagent (R711, Vazyme, Nanjing, China) with shaking. Furthermore, small intestine tissue frozen at −80 °C was placed on ice, melted to room temperature, sliced into small pieces, weighed (50 mg), added to grinding beads with 500 μL of FreeZol Reagent, and milled 2–3 times until there were no tissue clumps. The tissues were then kept for 5 min at room temperature, treated with Dilution Buffer (150 μL), shaken well, and left to stand for 5 min at room temperature. Then, the samples were centrifuged at 11,200 rpm (12,000× *g*) for 15 min at room temperature to precipitate the impurities. The supernatant aspirate (500 μL) was mixed with an equal volume of isopropanol, inverted, mixed, and left to stand for 10 min at room temperature. The sample was again centrifuged at 11200 rpm (12,000× *g*) for 10 min at room temperature to precipitate RNA and mixed with 1 mL of 75% ethanol. The precipitates were resuspended, centrifuged at 9100 rpm (8000× *g*) for 3 min at room temperature to collect the pellet. This step was repeated once again. Subsequently, the acquired sample was air-dried, mixed with 20 μL of RNase-free water, and gently pipetted 10 times to fully dissolve the RNA. Lastly, 1 μL of RNA was used to assess its concentration and purity.

### 4.14. RNA Reverse Transcription

The reaction system was prepared per the protocol provided in the reverse transcription kit (R333, Vazyme) (Table 4). The reverse transcription reaction conditions were as follows: reverse transcription at 50 °C for 15 min, followed by 85 °C for 5 s, and then maintained at 4 °C (Table 4). The entire protocol was performed on ice. Briefly, the reaction system was set up according to the instructions for the real-time fluorescence quantitative PCR kit (Q712, Vazyme) (Table 5). The reaction program is outlined in Table 6 (Step One Software™ Version 2.3).

### 4.15. Cellular ROS Assay

HIEC-6/IEC-6 cells were irradiated with 15 Gy γ, immediately treated with sDR5-Fc, and cultured for 24 h. Then, the medium was discarded, and the cells were washed twice with PBS. DCFH-DA (S0033S, Beyotime, Haimen, China) was diluted with serum-free culture solution (1:1000) to achieve a final concentration of 10 μmol/L. The cells were collected, suspended in diluted DCFH-DA, and incubated at 37 °C for 20 min with inverted mixing every 3–5 min to allow complete interaction between the probe and the cells. Cells were rinsed thrice with serum-free cell culture medium to remove DCFH-DA that had not entered the cells. For flow cytometry, cells were resuspended in PBS, filtered through a 400-mesh sieve, and analyzed using an excitation wavelength of 488 nm and an emission wavelength of 525 nm. The data were analyzed using FlowJo V10 software.

### 4.16. Superoxide Dismutase (SOD) and Malondialdehyde (MDA) Assays

After sDR5-Fc treatment for 24 h, the cells were collected for MDA (BC0025, Solarbio, Beijing, China) and SOD (A001-3, Nanjing Jiancheng Bioengineering Institute, Nanjing, China) analyses. The protocol provided by the kit was followed.

### 4.17. Haematoxylin and Eosin (HE) Staining and Scoring

The mouse colon tissues were dissected, fixed in 4% paraformaldehyde solution at room temperature, and cut into 3 pieces of similar size from the same location for each group. The tissues were submerged in paraffin, dewaxed, transferred to water, stained with hematoxylin, followed by eosin, dehydrated, and sealed on microscope slides. The samples were then analyzed under the microscope, imaged, and scored for colonic pathology. From each group, 3 colon specimens were selected, and images were acquired at 200× from two severely damaged fields of view per sheet, which were categorized into 4 grades according to the degree of colon damage. The tissues were scored in descending order of severity: 0 = intestinal tissue mucosal epithelium is intact and normal; 1 = Mucosal epithelial cell loss, mild edema, epithelial collapse limited to the apical mucosal epithelium or other lesions; 2 = mild necrosis; 3 = moderate necrosis, crypts easily visible; 4 = severe necrosis of mucosal epithelium with loss of epithelial structures.

### 4.18. Immunohistochemistry

Mouse colon tissues from each group were paraffin-embedded, sectioned, dewaxed, hydrated, and antigenically repaired, and placed in 3% hydrogen peroxide solution at room temperature in the dark for 25 min. The slides were then placed in PBS (pH = 7.4) in a decolorizing shaker and washed with shaking for 3 times, each time for 5 min. In the histochemical procedure, 3% BSA was used to uniformly coat the tissue and incubated for 30 min at room temperature. The sealing solution was removed, and the tissue was treated with the PBS-diluted primary antibodies against NLRP3 (GB114320, Servicebio, Wuhan, China), Caspase-1 (GB15383, Servicebio), and ASC (GB113966, Servicebio) and incubated horizontally in a humid chamber at 4 °C overnight. Then, the slides were immersed in PBS (pH = 7.4) on a decolorizing shaker, agitated, rinsed thrice for five minutes each, partially desiccated, and probed with a secondary antibody (HRP-labeled goat anti-rabbit IgG) at ambient temperature for 50 min. Tissues were treated with DAB for color development, re-stained with hematoxylin for 3 min, rinsed with running water, dehydrated, and sealed. Images were subsequently captured under the microscope.

### 4.19. Luminex Liquid Chip

Mice’s whole blood was collected by removing the eyeballs and collected in 1.5 mL sodium heparin anticoagulant tubes, centrifuged at 4 °C and 3000× *g* for 20 min to harvest supernatant, which was stored at −80 °C until use. Then, the standard, beads, mixed detection antibody, washing solution, and PE-streptavidin were prepared according to the kit’s instructions (PPX-03, Thermo, Waltham, MA, USA). The beads and sand were then added to the samples sequentially, as per the kit guide, at room temperature and at 800 r/min for 2 h. The sample volumes are presented in Table 7. Then, the beads were rinsed with 100~200 μL of washing solution 3 times, mixed with 25 μL of detection antibody at 800 r/min for 0.5~1 h at room temperature. The beads were rinsed again with 100~200 μL of washing solution 3 times, and mixed with 50 μL of PE-streptavidin at 800 r/min for 10~30 min at room temperature. Then, the beads were rinsed with 100~200 μL of washing solution 3 times, and added to 100~150 μL of rinse/sheath solution for 0.5~2 min at 800 r/min and room temperature. Lastly, the samples were analyzed on the machine.

### 4.20. Statistical Analysis

All statistical measurements were performed using GraphPad Prism 8.0.1, and all data are expressed as the mean ± standard error of the mean (SEM). The intergroup differences were assessed using one-way or two-way ANOVA. *p* < 0.05 was considered statistically significant.

## 5. Limitations

However, this study has some limitations. (1) The molecular mechanisms and signaling pathways through which DR5 modulates pyroptosis remain incompletely elucidated. (2) The cross-regulation between NLRP3 and Caspase-8 remains undetermined, and the latter is a critical component of the TRAIL/DR5 apoptotic pathway that can induce GSDMD cleavage and pyroptosis. (3) Further clarification is required on the exact mechanism of DR5 internalization and interaction with NLRP3 under irradiation conditions. (4) The molecular pathway by which sDR5-Fc mitigates oxidative stress-induced pyroptosis warrants further research. (5) Since the current evidence is mainly derived from molecular docking and recombinant-protein-based BLI assays, further genetic validations using DR5 or NLRP3 knockdown/knockout cell and animal models are required to firmly establish the functional DR5-NLRP3 axis in radiation-triggered intestinal pyroptosis. Future work including co-immunoprecipitation (co-IP) and proximity-ligation assay (PLA) in irradiated intestinal epithelial cells will help verify this protein–protein interaction in the cellular context. Whether radiation triggers DR5 internalization to access cytosolic NLRP3, or sDR5-Fc acts dominantly via neutralizing extracellular TRAIL to reduce upstream DISC assembly, remains to be clarified.

## 6. Conclusions

In summary, this study showed that a single high-dose (15 Gy) γ-ray irradiation induced significant oxidative stress and pyroptotic damage in intestinal epithelial cells. Furthermore, the DR5 antagonist, sDR5-Fc, can effectively inhibit the NLRP3/Caspase-1/GSDMD pyroptosis signaling pathway in irradiated intestinal epithelial cells. Moreover, sDR5-Fc treatment significantly alleviated radiation-induced intestinal injury in mice by reducing tissue damage, excessive oxidative stress, and pyroptosis, decreasing systemic inflammation levels, and improving post-radiation survival rates. Relative to amifostine, which necessitates preventative treatment, sDR5-Fc maintained therapeutic efficacy when given post-irradiation, underscoring its potential for addressing inadvertent radiation exposure. These findings suggest that DR5 is a crucial mediator of radiation-induced intestinal damage, and sDR5-Fc is a promising therapeutic candidate for radiation protection, warranting further comprehensive research.

## Figures and Tables

**Table 1:** Primer sequences used for RT-PCR (Human).

Gene	Forward Primer (5′-3′)	Reverse Primer (5′-3′)
*NLRP3*	GGGACTGAAGCACCTGTTGT	GAGTCTGGTCAGGGAATGGC
*Caspase-1*	ATCCGTTCCATGGGTGAAGG	GCCCCTTTCGGAATAACGGA
*GSDMD*	AGGACAGGCAAAGATCGCAG	CCAGGTGTTAGGGTCCACAC
*IL-1β*	CAGAAGTACCTGAGCTCGCC	AGATTCGTAGCTGGATGCCG
*IL-18*	TGGCTGCTGAACCAGTAGAAG	GAGGCCGATTTCCTTGGTCA
*β-actin*	AGCCTTCCTTCCTGGGCAT	GCCAGGGCAGTGATCTCCTT

**Table 2:** Primer sequences used for RT-PCR (Rat).

Gene	Forward Primer (5′-3′)	Reverse Primer (5′-3′)
*NLRP3*	CTCACCTCACACTCCTGCTG	GCCCTGCTGTTTCAGAACCT
*Caspase-1*	GACCGAGTGGTTCCCTCAAG	GACGTGTACGAGTGGGTGTT
*GSDMD*	TTTGCGGGAGTGGTCAAGAA	CGGGGTTTCCAGAACCATGA
*IL-1β*	GCAGCTTTCGACAGTGAGGA	CCCAAGTCAAGGGCTTGGAA
*IL-18*	GGCTGCCATACCAGAAGAAGG	CACAGGCGGGTTTCTTTTGT
*β-actin*	AGATCAAGATCATTGCTCCTCCT	ACGCAGCTCAGTAACAGTCC

**Table 3:** Primer sequences used for RT-PCR (Mouse).

Gene	Forward Primer (5′-3′)	Reverse Primer (5′-3′)
*NLRP3*	AACTTGCAGAAGCTGGGGTT	TCCAAGGGCATTGCTTCGTA
*Caspase-1*	CCCAGGCAAGCCAAATCTTTATC	TCCTGGAAATGTGCCATCTTCTTT
*GSDMD*	TTGCTGCGCTTTGTTCCATC	GCTGGGCTGGTCCTGTAAAA
*IL-1β*	GCTTCAGGCAGGCAGTATCA	AGTCACAGAGGATGGGCTCT
*IL-18*	CAACTTTGGCCGACTTCACTG	TCAGTCTGGTCTGGGGTTCA
*β-actin*	ACTATTGGCAACGAGCGGTT	AATGCCTGGGTACATGGTGG

**Table 4:** Reverse transcription systems.

Reagents	Volume (μL)
RNase-free ddH_2_O	11
5× All-in-one qRT SuperMix	4
Enzyme Mix	1
Template RNA	4
Total	20

**Table 5:** RT-PCR reaction system.

Reagents	Volume (μL)
2× Taq Pro Universal SYBR qPCR Master Mix	10
Primer Mix (10 μM)	0.8
Template cDNA	4
RNase-free ddH_2_O	5.2
Total	20

**Table 6:** RT-PCR reaction conditions.

Stages		Number of Cycles	Temperature (°C)	Time (s)
Stage 1	Holding stage	1	95	30
Stage 2	Cycling stage	8	95	15
			60	10
			72	30
Stage 3	Cycling stage	40	95	15
			60	10
			72	30
Stage 4	Melt Curve stage	-	95	15
			60	60
			95	15

**Table 7:** Luminex spreadsheet.

Reagents	Volume (μL)
Capture Beads Mix	50
Plasma Samples	25
Standard Mixes	25
Detection Antibody	25
Streptavidin–PE(SA-PE)	50
Rending Buffer	120

## Data Availability

The original contributions presented in this study are included in the article. Further inquiries can be directed to the corresponding authors.

## Abbreviations

DR5	Death receptor 5
TRAIL	TNF-related apoptosis-inducing ligand
sDR5-Fc	Soluble death receptor 5 fusion protein
RIII	Radiation-induced intestinal injury
IR	Ionizing radiation
TAI	Total abdominal irradiation
GSDMD	Gasdermin D
H&E	Hematoxylin and eosin
IF	Immunofluorescence
IHC	Immunohistochemical
IL	Interleukin
LDH	Lactate dehydrogenase
NLRP	NLR family pyrin domain containing
ROS	Reactive oxygen species
MDA	Malondialdehyde
qRT-PCR	Real-time fluorescent quantitative polymerase chain reaction
SEM	Scanning electron microscope
WB	Western blotting
